# Role of tailored sourdough fermentation in the flavor of wholegrain-oat bread

**DOI:** 10.1016/j.crfs.2024.100697

**Published:** 2024-02-12

**Authors:** Silvia Cera, Fabio Tuccillo, Antti Knaapila, Finlay Sim, Jessica Manngård, Katariina Niklander, Michela Verni, Carlo Giuseppe Rizzello, Kati Katina, Rossana Coda

**Affiliations:** a Department of Food and Nutrition, P.O. Box 66 (Agnes Sjöbergin Katu 2), University of Helsinki, FI-00014, Helsinki, Finland; b Department of Environmental Biology, “Sapienza” University of Rome, Piazzale Aldo Moro 5, 00185, Rome, Italy; c Helsinki Institute of Sustainability Science, Faculty of Agriculture and Forestry, University of Helsinki, Helsinki, Finland

**Keywords:** Sensory profile, Volatile compound, Lactic acid bacteria, Yeast, Microbial starter, Gluten-free bread

## Abstract

Sourdough technology has been known for its role in the improvement of texture, flavor, and quality of mainly wheat and rye-based breads for decades. However, little is reported about its use in the improvement of whole-grain oat bread, especially concerning flavor formation, which is one major consumer drivers. This study investigated the effects of sourdough obtained by different lactic acid bacteria and yeast starters consortia on the texture and flavor of 100% oat bread. Four different consortia were selected to obtain four oat sourdoughs, which were analyzed to assess the main features due to the different starter fermentation metabolism. Sourdoughs were added to breads as 30% dough weight. Bread quality was technologically monitored via hardness and volume measurements. Sourdough breads were softer and had higher specific volume. The sensory profile of sourdoughs and breads was assessed by a trained panel in sensory laboratory conditions, and the volatile profile was analyzed by HS-SPME-GC-MS. Sourdoughs were rated with higher intensities than untreated control for most of attributes, especially concerning sour aroma and flavor attributes. Sourdough breads were rated with higher intensities than control bread for sour vinegar flavor and total odor intensity, in addition they had richer volatile profile. Our results confirmed that sourdough addition can lead to an enhanced flavor, moreover, it demonstrated that the use of different consortia of lactic acid bacteria and yeast strains leads to the improvement of texture and altered sensory profile of whole-oat bread.

## Highlights

• Oat sourdoughs fermented by different microbial associations and related breads were studied.
• Volatile compounds and sensory analyses were performed.
• Sourdough breads had a richer volatile profile and a more distinct flavor than control bread.
• The association of lactic acid bacteria and yeast led to intense aroma and sourness of the bread.

## Graphical abstract

**Figure:**
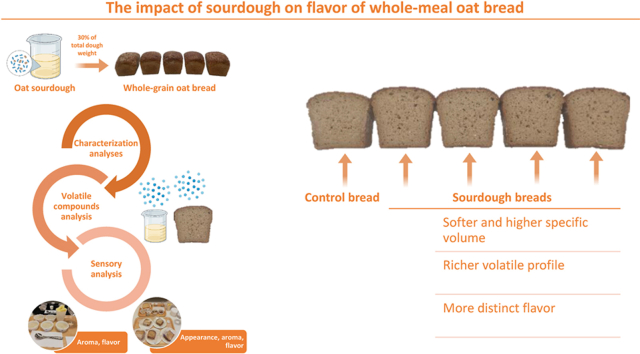

## Abbreviations

LAB: Lactic acid bacteria
fw: flour weight
dw: dry weight
WOF: whole grain oat flour
SPO: sprouted oat grain
T0C: untreated oat dough (time 0 control dough)
S1: *L. brevis* IC9 2% dw fructose sourdough
S2: *L. plantarum* 1MR20 sourdough
S3: *L. brevis* IC9 + *L. plantarum* 1MR20 sourdough
S4: *L. brevis* IC9 + *L. plantarum* 1MR20 + *S. cerevisiae* LNE10 sourdough
CB: control bread (sourdough is not added)
S1B: *L. brevis* IC9 2% dw fructose bread
S2B: *L. plantarum* 1MR20 bread
S3B: *L. brevis* IC9 + *L. plantarum* 1MR20 bread
S4B: *L. brevis* IC9 + *L. plantarum* 1MR20 + *S. cerevisiae* LNE10 bread
FQ: Fermentation quotient
TTA: total titratable acidity
HPLC: High-performance liquid chromatography
HS-SPME GC–MS: Headspace solid-phase micro extraction gas chromatography mass spectrometry
FAAs: free amino acids
SV: specific volume
VOCs: volatile organic compounds
PCA: principal component analysis
PC1: principal component 1
PC2: principal component 2
PLS: partial least squares regression analysis

## 1 Introduction

In the last few decades, the interest in using oats (*Avena sativa*) in food products has risen due to its excellent nutritional profile, the absence of gluten, and versatility for several plant-based applications (Mao et al., 2022). Oat is a source of dietary fiber, protein, unsaturated fatty acids, phenolic compounds, vitamins, and minerals (Joyce et al., 2019). Furthermore, the FDA (1997) and EFSA Panel on Dietetic Products Nutrition and Allergies (2011) have officially recognized, within the approval of health claims, the benefits of oat β-glucan against LDL-cholesterol and the risk of coronary disease. Moreover, oats do not contain gluten proteins, and this permits most celiacs to tolerate it if not contaminated by other gluten sources. However, the absence of gluten, the risk of lipid-derived off-flavors formation, and the distinct starch properties make the use of oat in food production challenging, especially for baked goods (Cappelli et al., 2020; Z. Yang et al., 2023). Most of the studies related to oat in baked products focused on the enrichment of wheat bread with oat to increase its fiber content, or on the application of enzymatic treatments/hydrocolloids to improve whole-oat bread texture (Flander et al., 2007, 2011; Y. Li et al., 2021; Renzetti et al., 2008, 2010; Saka et al., 2021). Indeed, the consumption of oat-based bread has been found beneficial for health due to cholesterol lowering and attenuation of blood sugar levels effects (Martínez-Villaluenga and Peñas, 2017; Momenizadeh et al., 2014); moreover, fermentation can confer to oat products an even higher potential to be defined as health-promoting food (Alemayehu et al., 2023).

Flavor has been defined as a “complex combination of the olfactory, gustatory and trigeminal sensations perceived during tasting” (ISO, 2008). In breadmaking, although the cereal flour used has peculiar aromatic characteristics, flavor formation of the final product is strongly affected by dough formulation, fermentation, lipid oxidation, and the baking process, including phenomena such as non-enzymatic Maillard reactions, caramelization and thermal degradation (Pétel et al., 2017; Pico et al., 2015). Nowadays, sourdough fermentation is a well-known technology used to enhance wheat and rye breads aroma (Pétel et al., 2017). In presence of sourdough, attributes, such as dairy sour, vinegar, lemon, and yeasty, have been previously used to describe wheat bread (Lotong et al., 2000). Sourdough addition strongly increased flowery yeasty and malty odor perceptions in the wheat crumb, while roasty aroma characterized the crust. Similarly, in rye bread, fermentation was able to enrich the sensory profile, however with different key odorants, such as the high acetic acid content in the crust (Callejo, 2011; Cho and Peterson, 2010; Grosch and Schieberle, 1997).

Baking with 100% oats is a technological challenge due to the lack of gluten network, leading to breads with reduced volume and high density. Optimization of the dough yield in oat bread was considered a critical parameter to reach good quality, with higher specific volume and softer crumb (Sammalisto et al., 2021). To overcome the absence of gluten-network in gluten-free products (Lynch et al., 2018), the addition of commercially available hydrocolloids and gums has been the most common strategy. However, the use of additives in gluten-free products is expensive and does not meet current consumer trend for clean label products. The pivotal role of sourdough in the improvement of wheat-bread nutritional properties, texture, palatability, loaf-specific volume, crumb softness, shelf-life, and flavor has been extensively demonstrated (Arora et al., 2021). The use of sourdough fermented by starter cultures selected based on their specific technological traits has improved the sensory, texture, and nutritional quality of gluten-free bread (Bender and Schönlechner, 2020; Galle et al., 2012; Gobbetti et al., 2008; Moroni et al., 2009; Rühmkorf et al., 2012; Šmídová and Rysová, 2022; Wolter et al., 2014b; Zhang et al., 2023b). Furthermore, in recent years, the interest in the changes induced by sourdough fermentation on the production of volatile organic compounds (VOCs) in gluten-free breads increased (Nissen et al., 2020).

Despite all these potential advantages, only a few studies focused on the role of oat sourdough to enhance 100% oat bread quality, resulting in higher specific volume and softer crumb (Hüttner et al., 2010) and, to the best of our knowledge, no studies have investigated the impact of oat sourdough on flavor formation of oat bread. Up to date, research about the sensory profile of breads containing oats focused mainly on composite wheat-oat blended breads (Flander et al., 2007; Ivanišová et al., 2023; Krochmal-Marczak et al., 2020), and only a few studies focused on whole-grain oat bread (Hager et al., 2012; Wolter et al., 2014a).

The major part of the oat flour currently used by the food industry is heat-treated (kilned) to inactivate lipase and peroxidase, responsible of the lipid derived off-flavors and rancidity during processing and storage (Z. Yang et al., 2019, 2023). This results in the typical appealing oat aroma and flavor, mainly due to the formation of Maillard-reaction end products (X. Li et al., 2022). However, heat treatment also leads to the inhibition of other endogenous enzymes, such as amylases and proteases which are known to play an important role in cereal fermentation (Gänzle et al., 2008).

Our aim was to study the influence of oat sourdough fermentation on flavor formation in oat bread. LAB species with different carbohydrate metabolism pathways i.e., homofermentative (*Lactiplantibacillus plantarum*) and heterofermentative (*Levilactobacillus brevis*), alone or in combination with the yeast *Saccharom*y*ces cerevisiae* most commonly representing the microbiota of sourdoughs (Martín-Garcia et al., 2023) have been used as starters to ferment wholegrain oat flour. The microbial quality, acidification, sugars, β-glucan content, viscosity and free amino acids of oat sourdoughs and their effects on the properties of 100% oat breads were determined. The sensory and volatile analyses of the oat sourdoughs and breads were performed, and the results were analyzed with multivariate methods to gain a more in-depth understanding of flavor formation during oat fermentation and baking.

## 2 Materials and methods

### 2.1 Raw materials and microbial starters

Heat treated whole grain coarse oat flour (WOF, Helsingin Mylly Oy, Järvenpää, Finland; nutritional composition per 100 g: 13 g of protein, 6.9 g of fat, 59 g of carbohydrates, 10 g of dietary fiber) and sprouted oat grain (SPO) (Sproutgrain oat, Puratos Estonia OÜ, Peetri, Estonia; 48% water) were used for sourdough making. Whole grain oat flour (as in the sourdough), gluten-free fine oat endosperm flour (Raisio Oyj, Raisio, Finland), tap water, fresh baker's yeast (Suomen Hiiva Oy, Lahti, Finland), sucrose (Suomen Sokeri Oy, Kantvik, Finland), and salt (Meira Oy, Helsinki, Finland) were used for bread making. A microbial screening for the best performances in oat sourdough led to the selection of LAB belonging to the following species: *Lactiplantibacillus plantarum* (strain 1MR20) and *Levilactibacillus brevis* (strain IC9) and the yeast *Saccharomyces cerevisiae* (strain LNE10) belonging to the culture collection of the University of Bari “Aldo Moro” (Bari, Italy) and available at the research collection of the Department of Food and Nutrition at the University of Helsinki (Helsinki, Finland) have been used as starters for WOF fermentation. LAB were routinely cultivated in MRS broth (Neogen®, Lansing, MI, USA). Before inoculum LAB were cultivated in microaerophilic condition at 30 °C for 24 h. When sourdoughs were made to be added to the breads, LAB were cultivated in general edible medium (GEM) containing 20 g dextrose, 20 g sucrose, 30 g soy peptone, 7 g yeast extract, 1 g MgSO4·7H2O in 1 L 0.01 M potassium phosphate buffer pH 6.3 at 30 °C for 24 h. The yeast was routinely cultivated in YPD broth (20 g of dextrose, 20 g of soy peptone, 10 g of yeast extract in 1 L) at 25 °C for 24 h. Dextrose, soy peptone, and yeast extract were purchased from Neogen® (Lansing, MI, USA), sucrose from Suomen Sokeri Oy (Kantvik, Finland) and MgSO_4_·7H_2_O from Merck (Darmstadt, Germany).

### 2.2 Sourdough fermentation

Sourdoughs were routinely prepared with a total weight of 150 g according to the recipe, which consisted of 30% WOF, 3.33% SPO, and 66.67% distilled water (dry ingredients: distilled water ratio 1:2). In S1 (fermented by *L. brevis* IC9), fructose (Suomen Sokeri Oy, Kantvik, Finland) replaced WOF being used as 2% of the total dry weight of the sourdough recipe (as 0.67% of 33.33%). Before inoculum, microbial cells were obtained from 24 h old cultures centrifuged at 10,000 rpm for 10 min and washed once in NaCl 0.9% w/v. Starters were inoculated into the sourdoughs alone (S1 = fermented by *L. brevis* IC9; S2 = fermented by *L. plantarum* 1MR20) or in combination (S3 = fermented by *L. brevis* IC9 + *L. plantarum* 1MR20; S4 = fermented by *L. brevis* IC9 + *L. plantarum* 1MR20 + *S. cerevisiae* LNE10) aiming at an initial cell density of ca. 6–7 Log CFU/g for LAB and of ca. 4–5 Log CFU/g for the yeast. Fermentation was conducted in triplicate at 30 °C for 24 h. Untreated oat dough (time 0 control dough = T0C) without addition of microbial starters was used as unfermented control in chemical and sensory analyses.

#### 2.2.1 Microbial cell counts

Cell counts were performed on raw materials, and before and after 24 h of fermentation on three biological replicates of sourdoughs according to Wang et al. (2022). Sourdoughs were serially diluted 1:10 in sterile saline solution (0.9% NaCl). LAB cell growth was confirmed with plate count method in MRS agar (Neogen®, Lansing, MI, USA), while yeast count was performed in YPD agar, having same recipe of YPD broth added with 1.5% no.1 bacteriological agar (Neogen®) and supplemented with 0.01% chloramphenicol (Neogen®). Agar plates were incubated at 30 °C for LAB and at 25 °C for yeast for 48–72 h. For presumptive *Enterobacteriaceae*, *Bacillus cereus* plate counts were performed on their respective substrates: VRGB (Neogen®, Lansing, MI, USA) and PEMBA (Lansing, MI, USA) (supplemented with 12.5 mL of Egg Yolk Emulsion and 5 mL of Polimixin B in 500 mL, Neogen®, Lansing, MI, USA). For *Enterobacteriaceae* and *Bacillus cereus*, plates were incubated at 37 °C for 24–48 h respectively.

#### 2.2.2 Determination of total titratable acidity (TTA), organic acids and sugars

Sourdough acidification was monitored before and after 24 h via pH measurements using a Mettler Toledo 340 pH meter (Leicester, UK). TTA was measured for three biological triplicates via an automatic pH titrator (Easy Plus, Mettler Toledo, Columbus, OH, USA) as described in Pöri et al. (2023). TTA was expressed as mL of NaOH 0.1 N needed to get 10 g of sourdough in 85 mL of Milli-Q water (Merck Millipore, Darmstadt, Germany), and 5 mL of technical grade acetone to a pH value of 8.5.

Lactic and acetic acids were quantified according to Xu et al. (2017) with some modifications. Four g of sourdough were diluted in 36 mL (16 mL for unfermented control doughs T0C) of Milli-Q water (Merck Millipore, Darmstadt, Germany); afterwards two centrifugations at 4,000 rpm for 15 min at 4 °C were performed to obtain a clearer supernatant. After a syringe filtration with 0.45 μm Acrodisc filters (Pall, USA). Analysis was performed with a Waters Alliance e2695 high-performance liquid chromatography (HPLC) system equipped with a photodiode array detector (PDA, Waters 996, Waters Corp., Milford, MA, USA), a refractive index detector (RI; Waters 2414, Waters Corp., Milford, MA, USA), and a Hi-Plex H column (300 × 6.5 mm; Agilent, CA, USA) with sulphuric acid (0.01 mol/L) as mobile phase. The column temperature was maintained at 65 °C and a flow rate of 0.6 mL/min was used. The quotient of fermentation (FQ) was calculated for all sourdoughs as the molar ratio between lactic acid and acetic acids.

Sugars were quantified in freeze-dried samples according to Xu et al. (2017), with some modifications. One hundred milligrams of freeze-dried samples were mixed with 5 mL of Milli-Q water and vortexed for 5 min to ensure complete dissolution of free sugars. The suspensions were boiled for 10 min enabling the inactivation of enzymes and microbes. After cooling, samples were centrifuged at 10,000 g for 10 min at 4 °C. Supernatant was collected in new flacon tubes and samples (500 μl) were filtered using Amicon® Ultra-0.5 mL centrifugal filter units (Merck Millipore Ltd., Cork, Ireland) at 10,000 g for 10 min. Samples were then diluted with Milli-Q water and injected (10 μl). Analysis was performed with a Waters Alliance e2695 HPLC system coupled with a Waters 2465 electrochemical detector (Waters, Milford, MA, USA). Sugars were separated by a CarboPac PA1 column (250 × 4 mm.id, Dionex, Sunnyvale, CA) with a flow rate of 1 mL/min. The gradient started at 2 mM NaOH for 3 min, then increased to 60 mM NaOH for 32 min. Column was then washed and regenerated with 200 mM NaOH. A post-column addition of 300 mM NaOH was applied to strengthen the signal. Glucose, fructose, sucrose, and maltose (Merck, Germany) were used as standards and 2-deoxy-D-galactose (Sigma-Aldrich) was used as the internal standard for quantification.

#### 2.2.3 β-glucan content and viscosity

β-glucan content was quantified on freeze-dried WOF and freeze-dried sourdoughs using a commercial kit (Mixed-Linkage β-glucan Assay kit) of Megazyme Ltd (Bray, Ireland). Viscosity was measured as described in Xu et al. (2017) with a few modifications. Measurements were conducted at 23 °C in triplicates for T0C after 1 h from mixing the ingredients and on sourdoughs after 24 h of fermentation using a rotational rheometer (Rheolaab QC, Anton Paar GmbH, Graz, Austria) equipped with a temperature device (C-PTD 180/AIR/QC), a ST22.02–4 V probe, and a measuring cup C-CC27/QC-LTD. Viscosity value at a shear rate of 100 s−1 was used to calculate the relative viscosity as the ratio between sourdough and control dough (T0C).

#### 2.2.4 Determination of free amino acids profile

Analysis of free amino acids (FAAs) was performed according to the methodology described in De Pasquale et al. (2021). The Biochrom 30+ series Amino Acid Analyzer (Biochrom, Cambridge Science Park, Cambridge, UK) with a Li-cation-exchange column of dimensions 4.6 × 200 mm was used to determine the concentration of FAAs, which were post-column derivatized by using ninhydrin reagent. Detection of FAAs was performed by absorbance at 440 (proline and hydroxyproline) or 570 nm (all the other amino acids).

### 2.3 Bread making

Bread formulations are reported in Table 1. The sourdoughs were added as 30.3% of the total dough weight. Breads were baked following the method described by Sammalisto et al. (2021). The ingredients were added to the bowl of a Varimixer (Metos Oy, Kerava, Finland) along with the sourdough (excluding CB) and mixed in the mixer using a paddle attachment for a total time of 7 min, starting at low speed (2) for 2 min and then at a higher speed (7) for 5 min. After resting for 15 min at room temperature, the dough was shaped by hand to make loaves of 500 g and placed in oiled baking pans (18 cm × 6 cm x 8 cm). The dough was proofed (using a Lillnord TopLine, Odder, Denmark) at 35 °C with 100% relative humidity for 30 min. A convection oven (Sveba Dahlen, Fristad, Sweden) was used to bake breads at 205 °C for 30 min, with 20 s of steaming before cooking. After baking, the breads were left to cool at room temperature for 1 h, then stored for analyses.

**Table 1:** Formulations of breads. Ingredients are indicated as % of flour basis (% fw), as % of dough weight (% dw). CB = control bread, no sourdough added; S1 = fermented by *L. brevis* IC9; S2 = fermented by *L. plantarum* 1MR20; S3 = fermented by *L. brevis* IC9 + *L. plantarum* 1MR20, and S4 = fermented by *L. brevis* IC9 + *L. plantarum* 1MR20 + *S. cerevisiae* LNE10.

	CB		S1B		S2B		S3B		S4B	
Ingredients	% fw	% dw	% fw	% dw	% fw	% dw	% fw	% dw	% fw	% dw
Water	90	45.45	51.23	25.25	51.02	25.25	51.02	25.25	51.02	25.25
Wholegrain oat flour	50	25.25	30.74	15.15	30.61	15.15	30.61	15.15	30.61	15.15
Endosperm oat flour	50	25.25	51.23	25.25	51.02	25.25	51.02	25.25	51.02	25.25
**Sourdough**	–	–	**61.47**	**30.3**	**61.22**	**30.3**	**61.22**	**30.3**	**61.22**	**30.3**
Salt	2	1	2.05	1.01	2.04	1.01	2.04	1.01	2.04	1.01
Sugar	3	1.5	3.07	1.52	3.06	1.52	3.06	1.52	3.06	1.52
Yeast	3	1.5	3.07	1.52	3.06	1.52	3.06	1.52	3.06	1.52
**Total**	**198**	**100**	**202.86**	**100**	**202.03**	**100**	**202.03**	**100**	**202.03**	**100**

#### 2.3.1 Bread characterization: specific volume, hardness, and TTA

Bread volume was measured according to Sammalisto et al. (2021) using a VolScan Profiler laser scanner (Stable Micro Systems Ltd, Godalming, UK). The specific volume (SV, mL/g) was calculated as loaf volume/loaf weight. The profile was measured on day 1, and on three loaves for each bread sample. The measurement of bread-crumb hardness was performed according to the methodology described in Sammalisto et al. (2021) with a few modifications to the test speed and rest time between the compressions. A texture profile analysis (two-bite compression test) was conducted using a TA-XT2i Texture Analyzer (Stable Micro Systems, Godalming, UK) after one day from baking. A 5 kg load cell and a P/36 R cylindrical probe were used. The bread crumb sample was placed under the probe and compressed twice into 40% deformation at 2 mm/s speed, with a 3 s rest between the compressions. TTA of breads was measured via an automatic pH titrator (Easy Plus, Mettler Toledo, Columbus, OH, USA) as described for the sourdoughs. Ten g of homogenized bread crumb were dissolved in 85 mL of Milli-Q water (Merck Millipore, Darmstadt, Germany) and 5 mL of acetone. TTA was expressed as mL of NaOH 0.1 N used by the titrator up to pH 8.5. The analyses mentioned above were conducted on three biological replicates.

### 2.4 Analysis of volatile compounds

Volatile profiling was performed on sourdoughs and breads. Headspace solid-phase micro extraction gas chromatography mass spectrometry (HS-SPME GC–MS) was used to analyze volatile organic compounds (VOCs) following the method described in Tuccillo et al. (2022a) with some modifications. Sourdoughs (T0C included) were frozen after 24 h of fermentation, thawed and mixed right before the sampling, while oat sourdough breads (CB included) were frozen after baking and thawed overnight at room temperature in plastic bags. Two g of sourdoughs (T0C included) or 2 g of homogenized sourdough bread (CB included) were transferred into 20-mL amber SPME vials. One biological replicate was analyzed in triplicate. The extraction was conducted using a 1 cm (50/30 μm phase thickness) divinylbenzene/carboxen/polydimethylsiloxane fiber (Supelco, Sigma Aldrich, St. Louis, MO, USA). After an incubation of 10 min, a 30 min extraction was performed with constant agitation speed of 250 rpm at 50 °C using different temperatures. VOCs were semi-quantified based on the relative area counts of the base peaks, which were manually integrated. Identification was conducted as reported in Tuccillo et al. (2022b) and was based on the correspondence of MS spectra and Linear Retention Indexes with available library entries (Wiley 7 N, Wiley Registry of Mass Spectral Data, 7th Edition; “Flavornet and human odor space” from Acree and Arn (2004) accessed on October 3, 2023) and published literature (Paradiso et al., 2009; Tuccillo et al., 2022b). LRI were determined by calculating the retention times of the alkane series 7–30 (Sigma Aldrich, St. Louis, MO, USA) and hexane (Sigma-Aldrich, Schnelldorf, Germany). Aroma descriptors used in the discussion section were obtained from “Flavornet and human odor” (Acree and Arn, 2004) and from Pétel et al. (2017).

### 2.5 Sensory evaluation

The sensory evaluation was conducted according to the methodology described in Wang et al. (2024). Eleven (3 males and 8 females) and 10 (4 males and 6 females) participants were trained for the evaluation of oat sourdoughs and oat breads, respectively, using generic descriptive analysis (GDA) carried out in the sensory laboratory, conforming to ISO8589. Recruitment of participants was done within the Department of Food and Nutrition of the University of Helsinki, and the selection was based on their previous experience in sensory evaluations. All participants were informed in advance about the aim of the study, possible allergens, and data treatment policy and they voluntarily participated by signing a written consent form. Panelists were requested not to drink or eat within 1 h before the sensory sessions. The study was conducted in accordance with the Declaration of Helsinki and ethical principles of sensory research conducted at our sensory laboratory approved by the University of Helsinki Ethical Review Board in Humanities and Social and Behavioral Sciences (Statement 15/2020).

#### 2.5.1 Oat sourdoughs and bread samples

Oat sourdoughs (T0C included) were evaluated after 24 h of fermentation after vigorously mixing to make the dough homogeneous, and around 10 g were served in plastic cups closed with a lid. For all sessions fresh sourdoughs and T0C were prepared. Oat sourdough breads (CB included) were frozen after baking and thawed overnight at room temperature in plastic bags. Breads were cut (1 cm thick) and 2 slices were served in closed plastic bags. All samples were presented with a 3-digit code.

#### 2.5.2 Training and sensory evaluations

The panel evaluated 13 attributes for sourdoughs (Table 2), 6 for aroma, and 7 for flavor. For breads, 11 attributes in total, which included 1 for appearance, 1 for aroma, and 9 for flavor (Table 2). To ensure consistency and accuracy, the panel underwent three training sessions for sourdoughs and two for breads, with each session lasting 1.5 h. These training sessions were dedicated to the careful selection of vocabulary and reference standards and the proficient use of the rating scale by anchoring to the scale with the reference standards.

**Table 2:** List of evaluated sensory attributes, definitions, references, and intensities for oat sourdoughs and oat breads (on a scale 0–10) following the order of evaluation.

Attribute (Sourdoughs)	Definition	Instruction	Reference	Intensity
Aroma		Leave the sample under the nose and immediately take a sniff when the lid is open. Put the lid back on the sample to write the score.		
Total Odor Intensity	Intensity of the whole odor experience		No reference	n/a
Raw Oat Aroma	Aroma of raw oat		Oat porridge (oat flakes + water)	9
Sour Aroma (Dairy)	Aroma of dairy sourness		Sour milk	9
Sour Aroma (Vinegar)	Aroma of vinegar, acetic acid		Vinegar solution 1:20	8
Fresh Yeast Aroma	Aroma of fresh yeast, bread dough		Fresh yeast slices	10
Malt Aroma	Aroma of malt		Oat malt extracted	9
Taste/Flavor		Take the sample with the spoon and place it in the mouth. Let it spread around the oral cavity and chew it for few seconds and then evaluate it. You can swallow or spit the sample.		
Total Flavor Intensity	Intensity of the whole flavor experience		No reference	n/a
Raw Oat Flavor	Flavor of raw oat		Oat porridge (oat flakes + water)	9
Sourness (Dairy)	Flavor of sour milk, dairy sourness		Sour milk	7
Sourness (Vinegar)	Flavor of vinegar		Vinegar solution 1:20	8
Lemon Flavor	Reminds lemon flavor		Lemon slices	9
Fresh Yeast Flavor	Flavor of fresh yeast, bread dough	You can sniff the reference of fresh yeast aroma just as a reminder.		n/a
Malt Flavor	Flavor of malt	You can sniff the reference of malt aroma just as a reminder.		n/a

Attribute (Breads)	Definition	Instruction	Reference	Intensity
Appearance				
Color of Crust	Intensity of crust color	Observe the color of crust for at least 5 s.	White wheat bread	7
Aroma		Take a sniff of the samples (containing crust and crumb) placing it close to the nose, almost touching it.		
Total Odor Intensity	Intensity of the whole odor experience		No reference	n/a
Taste/Flavor		Place only a piece of sample crumb into the mount and chew it for 10 s. Wait until taste occurs. After that you can swallow or spit the sample.		
Sourness (Dairy)	Sour taste of sour milk		Sour milk	7
Sourness (Vinegar)	Sour taste of vinegar		Vinegar solution 1:20	8
Sweetness	Typical sweet taste, like in table sugar		Sugar solution 2%	8
Oat Flavor	Characteristic flavor of oat		Oat porridge (oat flakes + water)	9
Yeast Flavor	Typical flavor of yeast, bread dough containing yeast			n/a
		Place a piece of sample containing crust and crumb into the mount and chew it for 10 s. Wait until taste occurs. After that you can swallow or spit the sample.		
Nutty Flavor	A slightly sweet brown, nut-like impression	For reference sample: place a piece of wheat bread crumb and a hazelnut into the mouth, chew for 10 s and wait until the flavor occurs.	Wheat bread crumb + Hazelnuts	10
Total Flavor Intensity	Intensity of the whole flavor experience		No reference	n/a
Overall Intensity of Aftertaste	Intensity of the aftertaste if present	Take one piece of sample (crust and crumb) and chew it for 5 s. Swallow it or spit it. Wait at least 30 s until aftertaste occurs before assigning a score.	No reference	n/a
Toasted Flavor	A brown, burnt, baked aromatic that may occur with grain	Place only a piece of sample crust into the mount and chew it for 10 s. Wait until taste occurs. After that you can swallow or spit the sample.	Rye chips salted	9

*n/a = no reference standard was used.

Three evaluation sessions (replicates) were performed by the panel to evaluate the intensity of each attribute on a line scale from 0 (not at all) to 10 (very strong) using the reference standards. Attributes were evaluated following the same order reported in Table 2. The order of sourdough and bread samples was randomized for each participant and between the three evaluations via block design (FIZZ © Version 2.51, Biosystèmes, Couternon, France).

### 2.6 Statistical analysis

The results of microbiological, chemical and texture profile analyses of sourdoughs and breads are reported as average of three replicates, except for free amino acid analysis (n = 2). The software used was IBM SPSS Statistics (Version 29, IBM®, Chicago, IL, USA) and SIMCA® (Version 15, Sartorius Corporate Administration, Göttingen, Germany) for univariate and multivariate analysis, respectively. Statistical analyses were conducted at a significance level of *p* < 0.05. The methodology described in Tuccillo et al. (2022a) was followed to assess the normality distribution, the panel performance, the homogeneity of variances and to conduct the multivariate analysis.

A three-way analysis of variance (ANOVA) was conducted to assess the panel performance by interpreting the significance values of the interaction effects (sample, replicate, participant, sample*replicate, sample*participant, replicate*participant). The study focused on main effects and two-way interactions. The analysis was performed by setting Sample as fixed factor, and Participant and Replicate as random factors. In total, 165 (11 Participants*3 Replicates*5 Samples) and 150 (10 Participants*3 Replicates*5 Samples) observations for each sensory attribute from sourdough and bread sensory analyses, respectively, were analyzed.

Kolmogorov-Smirnova (with Lilliefors significance correction) and Shapiro-Wilk tests were conducted to assess the normality of data distribution. Skewness test was used to assess the symmetry, whereas kurtosis to investigate pointiness of data distribution, two main methods to evaluate how much data deviate from normal (Ghasemi and Zahediasl, 2012). Skewness and kurtosis values ranging from −1 to 1 defines an approximate normal distribution (Mishra et al., 2019). Levene's test was performed to assess the homogeneity of variances for each attribute by using all participants' observations on all samples for the three evaluations. ANOVA values were replaced by the Welch test when Levene's test detected inhomogeneous variances. The significant differences were explored via the Tukey Test and checked via the Kruskal Wallis test with pairwise comparison (Bonferroni correction applied); however, no major differences were found.

For multivariate analysis, average measurements data was used and UV-scaled (unit variance). A principal component analysis (PCA) was performed to explore relationships of VOCs in sourdoughs and breads, both separately and together. For sourdoughs (n = 5), 29 VOCs were included, whereas for breads (n = 5), 39 VOCs. For the PCA of sourdough and breads (n = 10), 42 VOCs were included. A partial least squares regression analysis (PLS) was used to investigate the relationship between volatile compounds and sensory attributes. For the PLS model of sourdoughs (n = 5), 29 VOCs were reported (x), and 13 sensory attributes (y), whereas for the PLS of breads (n = 5), 39 VOCs (x), and 10 sensory attributes (y). Color of crust was removed from the PLS model of breads because of low relevance in the model.

## 3 Results

### 3.1 Sourdough characterization: microbial cell density, acidity, sugars, β-glucan and viscosity

LAB, yeasts, *Enterobacteriaceae* and *B. cereus* were not detected in the WOF. LAB were found in the SPO at 7.1 log CFU/g, while yeasts, *Enterobacteriaceae* or *B. cereus* were not detected. Before fermentation, S1, S2, S3 and S4 had a LAB cell density of 7.0 ± 0.1, 7.3 ± 0.1, 7.1 ± 0.03, and 7.2 ± 0.04 log CFU/g respectively. After 24 h of fermentation at 30 °C, in S1, S2, S3, and S4 LAB cell density was 8.9 ± 0.1, 9.7 ± 0.1, 9.7 ± 0.2, and 9.6 ± 0.1 log CFU/g, respectively. S4 had an initial yeast cell density of 4.4 ± 0.1 log CFU/g, which increased to 6.2 ± 0.1 log CFU/g after fermentation. Acidification, fermentation quotient and free sugars data are shown in Table 3. All sourdough combinations reached a pH value ca. 3.9. The combination S3 reached the lowest pH value of 3.85, while the highest value of 3.98 was found in S4 sourdough. TTA values after fermentation ranged from 8.1 to 9.1 mL NaOH, with S3 as the most acidic sourdough. Lactic acid was the predominant organic acid produced (5.45–7.08 mg/g) whereas acetic acid was detected only in traces in all sourdoughs except S1 (0.64 mg/g). The fermentation quotient (FQ, molar ratio between lactic acid and acetic acid) ranged from 5.8 to 33.6, with the lowest value found for S1. Free sugar profile significantly changed after fermentation, except for maltose (ca. 0.3%) which was similar in both T0C and S1 sourdough. Glucose was significantly different after fermentation in all sourdoughs, decreasing from 0.15% to 0.01–0.02% dry weight. Maltose significantly decreased from 0.3 to 0.03–0.04% dw in S2, S3 and S4, while it remained stable in S1. Sucrose was 0.91% dw in unfermented control dough, and it was not detected in any of the sourdoughs, except for S1, still containing 0.28% dry weight. Finally, fructose was 0.03% dw in T0C and was not found after fermentation. The exception was S1, which originally contained 2% dw of fructose before fermentation and 0.1% dw after fermentation. β-glucan content was approximately 4% dw of T0C and sourdoughs, and its content did not decrease after fermentation. The relative viscosity calculated as explained in 2.2.3 for all sourdoughs varied from 1.67 to 1.77 (sourdough viscosity Pa·s/T0C viscosity in Pa·s), while viscosity was 4.7 Pa s at the moment of mixing the ingredients therefore, an increase of viscosity (ranging from 7.8 to 8.5 Pa s) was detected in all sourdoughs.

**Table 3:** pH, TTA, organic acids values and related fermentation quotient (FQ) and sugars. Lactic acid and acetic acid are indicated as mg of acid/g of dough, in t0 control and after 24 h of fermentation in all sourdoughs. Free sugars are reported as percentage (%) of dry weight (dw). The data were reported as means of three biological replicates ± standard deviation. Different superscript lowercase letters (a-c) in the same column indicate statistically significant (Tukey's, *p* < 0.05) differences (n = 3); nd = not detected.

Sample	pH	TTA (mL of NaOH)	Lactic acid (mg/g)	Acetic Acid (mg/g)	FQ	Glucose (% dw)	Sucrose (% dw)	Fructose (% dw)	Maltose (% dw)
T0C	6.06 ± 0.01^c^	1.6 ± 0.13^a^	0.36 ± 0.07^a^	nd	–	0.15 ± 0.01^b^	0.91 ± 0.01^b^	0.03 ± 0.02^a^	0.33 ± 0.03^b^
S1	3.96 ± 0.03^b^	8.64 ± 0.2^bc^	5.55 ± 0.18^bc^	0.64 ± 0.03^c^	5.79	0.02 ± 0.01^a^	0.28 ± 0.11^a^	0.1 ± 0.03^b^^a^	0.3 ± 0.13^b^
S2	3.89 ± 0.03^a^	8.8 ± 0.15^bc^	7.03 ± 0.55^bc^	0.15 ± 0.04^a^	33.61	0.01 ± 0.00^a^	nd	nd	0.03 ± 0.01^a^
S3	3.85 ± 0.02^a^	9.07 ± 0.33^c^	7.08 ± 0.65^c^	0.20 ± 0.03^a^	24.65	0.01 ± 0.01^a^	nd	nd	0.04 ± 0.02^a^
S4	3.98 ± 0.03^b^	8.11 ± 0.53^b^	5.45 ± 1.01^b^	0.37 ± 0.04^b^	9.87	0.02 ± 0.01^a^	nd	nd	0.04 ± 0.01^a^

a In S1 fructose was added as 2% of dw.

#### 3.1.1 Free amino acids profile

As shown in Table 1 from Appendix A (Table A1), before fermentation (T0C), the total amino acids content was 3030.4 mg/kg. In contrast, total FAAs content in all sourdoughs after fermentation ranged from 1311.6 to 1454.9 mg/kg, with the highest value in S1 and the lowest amount in S4. The individual FAA concentration in T0C and sourdoughs are also indicated. Most of the FAAs decreased during fermentation; however, a slight increase was detected, especially in S1 and S4, for glycine, cysteine, GABA, ammonia and ornithine, which increased moderately in S1 (80.5, 55.3, 147.5, 135.1, and 48.2 mg/kg respectively). GABA, ammonia and ornithine were detected in higher concentrations in S3 than T0C (120.9, 84.3, 113.02 mg/kg respectively). Finally, proline increased in S4 (187.1 mg/kg).

### 3.2 Bread characterization: specific volume, hardness, and total titratable acidity (TTA)

All sourdough breads had higher SV than control bread (Table 4). Specific volume (SV) of the four sourdough breads obtained was significantly different and higher than the control bread SV (1.66–1.68 vs. 1.5 mL/g). No significant differences were found between sourdough breads. Bread crumb hardness on day 1 was significantly higher in control bread (30.6 N) than in sourdough breads (24.5–26.3 N). S1B and S3B were the softest breads, with 24.5 N and 24.7 N, respectively (Table 4). The total titratable acidity of breads showed a significant difference between CB with 2.7 mL and sourdough breads ranging from 5.2 to 5.7 mL of NaOH (Table 4).

**Table 4:** Specific volume (mL/g), hardness (N), and total titratable acidity (NaOH mL) of CB and sourdough breads. Data are reported as mean and standard deviation of three biological replicates (n = 3). Different superscript lowercase letters (a-b) in the same row indicate statistically significant (Tukey's, *p* < 0.05) differences.

	CB	S1B	S2B	S3B	S4B
**Specific volume (mL/g)**	1.5 ± 0.01^a^	1.68 ± 0.02^b^	1.66 ± 0.01^b^	1.68 ± 0.03^b^	1.67 ± 0.03^b^
**Hardness (N)**	30.56 ± 1.19^b^	24.52 ± 0.34^a^	26.31 ± 0.57^a^	24.70 ± 0.38^a^	25.27 ± 2.62^a^
**Total titratable acidity (NaOH mL)**	2.74 ± 0.09^a^	5.57 ± 0.16^bc^	5.44 ± 0.12^bc^	5.66 ± 0.17^c^	5.22 ± 0.23^b^

### 3.3 Volatile compounds profile of sourdoughs and breads

VOCs were analyzed to characterize flavor-active molecules in oat sourdoughs and breads (Table 5). Alcohols, aldehydes, carboxylic acids, esters, furans, pyrazines, terpenes, and organic compounds classes were detected. However, most of the compounds belonged to the alcohols, aldehydes, and carboxylic acids. Ethanol, acetic acid, 3-methylbutanol, 2-methylbutanol, 1-hexanol, hexanal, and nonanoic acid were the most prevalent VOCs found in all samples analyzed. In T0C fewer compounds than in sourdoughs were detected generally with low peak areas. In sourdoughs ethanol, acetic acid, 1-hexanol, and hexanal were the main compounds. In S2 and S3, the highest peak areas were reached by acetic acid, 1-hexanol, hexanal, and for only S2 also acetoin, whereas in S1 acetic acid, ethanol, 1-hexanol, hexanal, nonanoic acid, and 3-methylbutanol. S4 had the richest profile among the sourdoughs with prevalent amounts of ethanol, acetic acid, 3-methylbutanol, 1-hexanol, 2-methylbutanol, nonanoic acid, and hexanal.

**Table 5:** Volatile compounds detected via HS-SPME GC–MS and relative peak area (A) and standard deviation (SD). “/” is for not detected.

						T0C		CB		S1		S1B		S2		S2B		S3		S3B		S4		S4B	
	CAS	RT (min)	mLRI	rLRI	ID	A	SD	A	SD	A	SD	A	SD	A	SD	A	SD	A	SD	A	SD	A	SD	A	SD
**Alcohols**																									
1-Heptanol	111-70-6	21.4	1025	1210	LRI, MS	/	/	/	/	0.010	0.005	/	/	0.007	0.004	0.003	0.001	0.006	0.004	/	/	0.013	0.011	0.003	0.001
1-Hexanol	111-27-3	17.8	924	920	LRI, MS	0.015	0.008	0.036	0.009	0.189	0.096	0.041	0.032	0.182	0.115	0.069	0.043	0.116	0.121	0.044	0.039	0.266	0.215	0.062	0.043
1-Pentanol	71-41-0	14.1	823	822	LRI, MS	0.033	0.013	0.014	0.003	0.048	0.024	0.013	0.010	0.031	0.020	0.021	0.013	0.028	0.029	0.013	0.011	0.036	0.030	0.019	0.014
1-Propanol	71-23-8	6.7	616	627	LRI, MS	/	/	0.002	0.000	/	/	0.002	0.001	/	/	0.004	0.000	/	/	0.001	0.000	/	/	0.002	0.002
2-Ethyl-1-hexanol	104-76-7	22.2	1048	1123	LRI, MS	/	/	0.004	0.002	/	/	0.005	0.003	/	/	0.007	0.005	/	/	0.006	0.005	/	/	0.006	0.004
2-Methyl-1-propanol	78-83-1	8.9	685	/	MS	/	/	0.025	0.006	/	/	0.027	0.023	/	/	0.035	0.023	/	/	0.013	0.007	0.029	0.025	0.028	0.018
2-Methylbutanol	137-32-6	13.0	795	846	LRI, MS	0.005	0.000	0.055	0.012	0.063	0.032	0.055	0.043	0.028	0.021	0.078	0.050	0.028	0.030	0.037	0.027	0.205	0.169	0.073	0.050
2-Propyldecan-1-ol	60,671-35-4	30.1	1300	/	MS	/	/	0.003	0.001	/	/	0.003	0.003	/	/	0.004	0.002	/	/	0.004	0.004	/	/	0.004	0.003
3-Methylbutanol	123-51-3	12.9	792	813	LRI, MS	0.020	0.009	0.145	0.046	0.101	0.041	0.155	0.120	0.009	0.004	0.223	0.142	0.017	0.019	0.103	0.072	0.400	0.330	0.181	0.122
Ethanol	64-17-5	4.2	n/a	759	MS	0.117	0.056	0.815	0.170	0.445	0.230	0.933	0.735	0.058	0.037	1.169	0.741	0.081	0.110	0.578	0.410	1.569	1.311	1.048	0.713
Phenylethyl Alcohol	60-12-8	27.4	1208	1272	LRI, MS	/	/	0.006	0.001	/	/	0.009	0.008	/	/	0.011	0.007	/	/	0.007	0.007	0.016	0.015	0.012	0.009
**Aldehydes**																									
2-Heptanal	18,829-55-5	21.3	1022	1005	LRI, MS	/	/	0.002	0.000	/	/	0.006	0.004	0.009	0.006	0.007	0.004	0.003	/	0.005	0.004	0.009	/	0.006	0.004
2-Methylbutanal	96-17-3	9.6	706	729	LRI, MS	/	/	0.004	0.001	/	/	/	/	/	/	/	/	/	/	/	/	/	/	/	/
2-Octenal	2363-89-5	24.8	1126	1115	LRI, MS	0.015	0.005	0.013	0.005	0.058	0.037	0.016	0.012	0.049	0.036	0.026	0.016	0.030	0.028	0.017	0.015	0.052	0.043	0.020	0.015
Benzaldehyde	100-52-7	21.8	1035	1051	LRI, MS	/	/	0.008	0.001	0.015	0.006	0.008	0.006	0.013	0.010	0.011	0.007	0.009	0.009	0.008	0.008	0.009	0.007	0.009	0.006
Heptanal	111-71-7	18.7	949	939	LRI, MS	/	/	0.009	0.002	0.008	0.003	0.012	0.009	0.003	/	0.016	0.010	0.005	0.003	0.010	0.009	0.015	/	0.019	0.018
Hexanal	66-25-1	14.9	845	840	LRI, MS	0.158	0.052	0.151	0.030	0.176	0.105	0.163	0.127	0.192	0.136	0.202	0.126	0.136	0.137	0.140	0.123	0.103	0.088	0.149	0.097
Nonanal	124-19-6	25.8	1156	1152	LRI, MS	0.023	0.003	0.029	0.007	0.048	0.029	0.033	0.026	0.047	0.034	0.041	0.026	0.043	0.042	0.033	0.031	0.047	0.040	0.042	0.031
Pentanal	110-62-3	10.9	741	734	LRI, MS	0.009	0.002	0.010	0.003	/	/	0.018	0.014	0.004	0.002	0.023	0.014	0.005	0.005	0.013	0.011	/	/	0.016	0.011
**Carboxylic acids**																									
2-Methylbutanoic acid	116-53-0	18.5	942	1040	LRI, MS	/	/	/	/	/	/	0.015	0.011	/	/	0.020	0.012	/	/	0.011	0.008	/	/	0.015	0.011
3-Methylbutanoic acid	503-74-2	18.2	935	1040	LRI, MS	/	/	0.004	0.001	/	/	0.023	0.018	0.002	0.001	0.034	0.021	0.001	0.001	0.018	0.015	0.013	0.006	0.022	0.016
Acetic acid	64-19-7	9.7	707	752	LRI, MS	0.014	/	0.017	0.002	0.852	0.705	0.087	0.069	0.483	0.329	0.088	0.057	0.320	0.301	0.056	0.048	0.525	0.455	0.086	0.062
Butyric acid	107-92-6	16.3	882	1000	LRI, MS	/	/	0.004	0.000	/	/	0.011	0.009	/	/	0.017	0.011	/	/	0.010	0.008	/	/	0.013	0.010
Hexanoic acid	142-62-1	22.9	1069	1186	LRI, MS	0.005	/	0.004	0.001	0.052	0.034	0.009	0.008	0.034	0.022	0.013	0.008	0.027	0.026	0.009	0.007	0.035	0.030	0.013	0.011
Isobutyric acid	79-31-2	15.2	854	/	MS	/	/	0.011	0.004	/	/	0.041	0.032	/	/	0.063	0.040	/	/	0.032	0.024	0.011	/	0.044	0.031
Nonanoic acid	112-05-0	31.7	1357	1366	LRI, MS	0.025	/	0.004	0.002	0.122	0.095	0.015	0.012	0.057	0.031	0.018	0.012	0.037	0.026	0.009	0.008	0.115	0.091	0.013	0.011
Octanoic acid	124-07-2	28.9	1260	1370	LRI, MS	/	/	/	/	0.019	0.012	/	/	0.012	0.007	/	/	0.006	0.005	/	/	0.019	0.016	/	/
**Esters**																									
Ethyl acetate	141-78-6	7.6	643	664	LRI, MS	0.036	0.015	0.001	/	0.026	0.012	0.002	0.002	0.012	0.006	0.003	/	0.024	0.026	0.001	/	0.043	0.036	0.001	0.001
Ethyl hexanoate	123-66-0	21.5	1028	1054	LRI, MS	/	/	/	/	0.013	0.004	/	/	/	/	/	/	/	/	/	/	0.023	0.020	/	/
Ethyl lactate	97-64-3	15.7	868	889	LRI, MS	/	/	/	/	0.007	0.003	/	/	/	/	/	/	0.004	0.004	/	/	0.052	0.043	/	/
**Furans**																									
2-Pentylfuran	3777-69-3	21.1	1014	1003	LRI, MS	0.010	/	0.028	0.009	0.016	/	0.028	0.022	0.009	0.007	0.037	0.023	0.007	0.002	0.027	0.025	0.013	/	0.030	0.020
5-Methyl-2-furfural	620-02-0	22.1	1045	1069	LRI, MS	/	/	/	/	/	/	0.010	0.008	/	/	0.011	0.007	/	/	0.009	0.008	/	/	0.009	0.007
Furfural	98-01-1	17.2	908	965	LRI, MS	/	/	0.006	0.001	/	/	0.042	0.033	/	/	0.048	0.029	/	/	0.039	0.037	/	/	0.030	0.020
Furfuryl alcohol	98-00-0	18.4	938	942	LRI, MS	/	/	/	/	/	/	0.006	0.005	/	/	0.006	0.004	/	/	0.005	0.004	/	/	0.007	0.005
**Pyrazines**																									
2,5-Dimethylpyrazine	123-32-0	18.9	954	996	LRI, MS	/	/	0.006	/	/	/	0.002	/	/	/	0.001	/	/	/	/	/	/	/	0.013	0.005
2-Methylpyrazine	109-08-0	15.7	868	919	LRI, MS	/	/	0.008	0.001	/	/	0.013	0.010	/	/	0.015	0.009	/	/	0.011	0.010	/	/	0.013	0.008
**Terpenes**																									
Alpha-pinene	80-56-8	18.9	952	945	LRI, MS	/	/	0.025	0.011	/	/	0.027	0.023	/	/	0.036	0.024	/	/	0.022	0.019	/	/	0.018	0.009
Delta-3-carene	13,466-78-9	21.7	1031	1056	LRI, MS	0.008	0.001	0.042	0.011	0.041	0.026	0.043	0.034	0.047	0.034	0.061	0.039	0.031	0.030	0.043	0.039	0.034	0.029	0.050	0.034
Limonene	138-86-3	22.4	1052	1056	LRI, MS	/	/	0.010	0.002	/	/	0.011	0.009	0.006	0.005	0.017	0.011	0.003	0.003	0.011	0.010	0.006	0.006	0.015	0.011
**Others**																									
3,5-Octadien-2-one	38,284-27-4	26.2	1168	1193	LRI, MS	/	/	0.003	0.001	/	/	0.003	0.003	/	/	0.004	0.003	/	/	0.003	0.003	/	/	0.004	0.003
Acetoin	513-86-0	12.6	786	809	LRI, MS	/	/	0.026	0.005	/	/	0.044	0.034	0.116	0.072	0.079	0.050	0.006	0.007	0.037	0.029	0.011	/	0.058	0.041
Dodecane	112-40-3	27.2	1200	1200	LRI, MS	/	/	0.013	0.003	/	/	0.014	0.010	0.007	/	0.019	0.012	/	/	0.014	0.013	/	/	0.017	0.012

RT, retention time. mLRI, measured Linear Retention Index. rLRI, reference Linear Retention Index. ID, identification method./, missing value. n/a = not applicable. rLRI were obtained from Paradiso et al. (2009), Tuccillo et al. (2022b) and “Flavornet and human odor space” (http://www.flavornet.org/flavornet.html) (accessed on October 3, 2023). The latter reports LRI of compounds chromatographically separated with the column OV17, having with similar polarities of the one used in the experiments and in the first two cited works (SPB-624).

The VOCs detected in breads revealed changes that occurred during proofing and baking. In CB, the most relevant compounds detected were ethanol, hexanal, and 3-methylbutanol. S2B, followed by S4B, had the richest profile among breads. Although acetic acid was present in sourdoughs, the area detected in all breads was extensively reduced. In the presence of sourdough, the ethanol peak increased in all breads, except for S3B. The highest value of ethanol was found in S2B. The peak of hexanal slightly increased or remained stable in all sourdoughs, except for S3B. The peak area of 3-methylbutanol increased in S1B, S2B, and S4B, in comparison with CB, while S3B had a lower peak area. Among typical compounds of the Maillard reaction, furfural, and 2-pentylfuran were detected in all breads. Principal component analysis (PCA) was performed for sourdoughs and breads to explore the relationships in the data of VOCs detected.

**Fig. 1:**
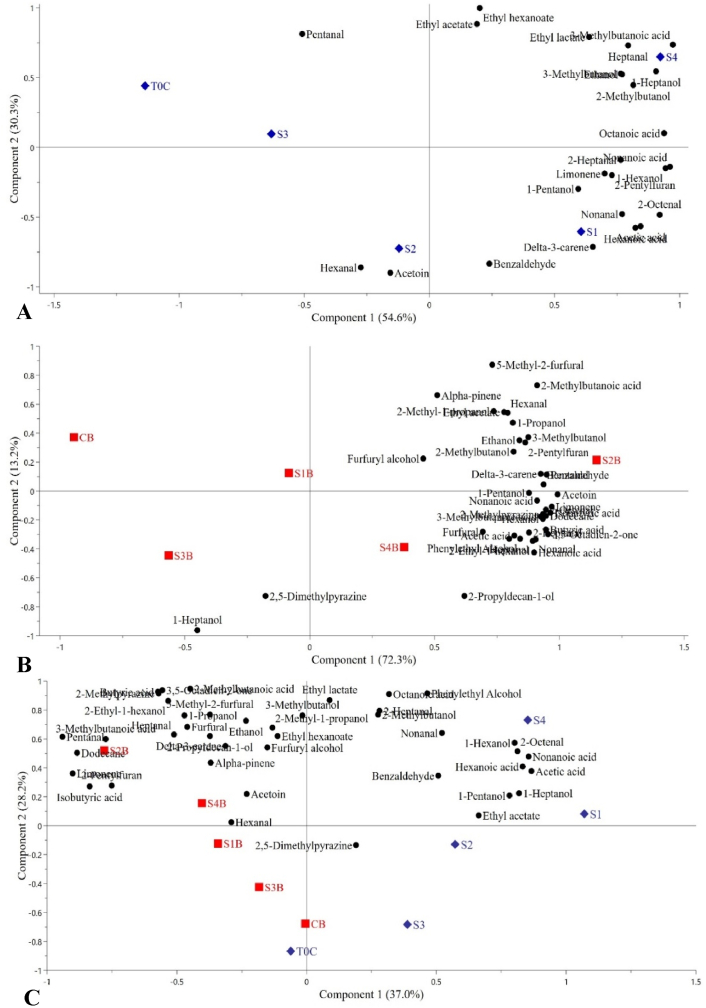
Principal Component Analysis (PCA) biplots of the average relative abundance of volatile compounds in sourdoughs only (A), and breads only (B), and sourdoughs and breads (C). Scores are shown as blue diamonds (sourdoughs) and red squares (breads). Loadings of the volatiles are shown as black dots. Principal components 1 and 2 are shown as x- and y-axes, respectively. The percentage in parentheses next to the axis label indicates the total variance explained by the component. T0C = untreated oat dough; S1 = fermented by *L. brevis* IC9; S2 = fermented by *L. plantarum* 1MR20; S3 = fermented by *L. brevis* IC9 + *L. plantarum* 1MR20, and S4 = fermented by *L. brevis* IC9 + *L. plantarum* 1MR20 + *S. cerevisiae* LNE10. CB = control bread, no sourdough added; “B” which follows the sourdough names indicates “bread”, e.g., S1B is the bread containing S1 sourdough. (For interpretation of the references to color in this figure legend, the reader is referred to the Web version of this article.)

In the PCA performed on sourdough data (Fig. 1, A), principal component 1 (PC1) and principal component 2 (PC2) explained 54.6% and 30.3% of the total variance, respectively. S4 and S1 were on the right side of PC1, whereas S2, S3, and finally T0C on the left side (PC1). A link between S4 and several VOCs, including heptanal, 3-methyl butanoic acid, ethanol, 1-heptanol, 2- and 3-methylbutanol, and ethyl lactate was found. Nonanal, acetic acid, hexanoic acid, delta-3-carene, 2-octenal, 1-pentanol, benzaldehyde, limonene, and 2-pentyl furan were associated with S1. Hexanal and acetoin were associated with S2, while S3 and T0C were not linked to most of the volatiles, except for pentanal. Regarding bread data (Fig. 1, B), PC1 and PC2 explained 72.3% and 13.2% of the total variance, respectively. S2B and S4B were on the right side of PC1, whereas S1B, S3B, and CB were on the left side (PC1). Although S2 did not have several connections with VOCs, PCA showed that the bread containing *L. plantarum* sourdough (S2B), followed by the consortium of LAB and yeast sourdough bread (S4B), had the richest profile having the most numerous connections with VOCs and overall higher peak area. S2B was linked to several VOCs, including 2-pentylfuran, benzaldehyde, pentanal, delta-3-carene, 2- and 3-methylbutanol, ethanol, 1-propanol, and hexanal. S4B was associated with several VOCs, including acetic acid, furfural, phenylethyl alcohol, heptanal, nonanal, 2-heptanal, 2-ethyl-1-hexanol, hexanoic acid, butyric acid, 3,5-octadien-2-one. In the PCA performed on sourdough and bread data together (Fig. 1, C), PC1 explained 37% of the total variance, whereas PC2 explained an additional 28.2%. While breads were on the left side of PC1, sourdoughs were placed on the right side. S2B and S4 were the samples with the most numerous relationships with VOCs, whereas CB and T0C were the samples with the least number of connections.

### 3.4 Sensory profile of sourdoughs and breads

Panel performance (Table A2), distribution of data, and the homogeneity of variances (Table A3) are reported in detail in Appendix A. Briefly, panelists agreed on the discrimination among the samples for most attributes, except for malt aroma (for sourdoughs), total odor intensity, yeast and nutty flavors, total flavor intensity, and overall intensity of aftertaste (for breads). Samples were consistent across replicates; however, the agreement of the panel through the sessions was not consistent. As common in descriptive sensory analysis, data deviated from a normal distribution for most attributes. In addition, variances were inhomogeneous for eight attributes of sourdoughs and for one of breads.

**Fig. 2:**
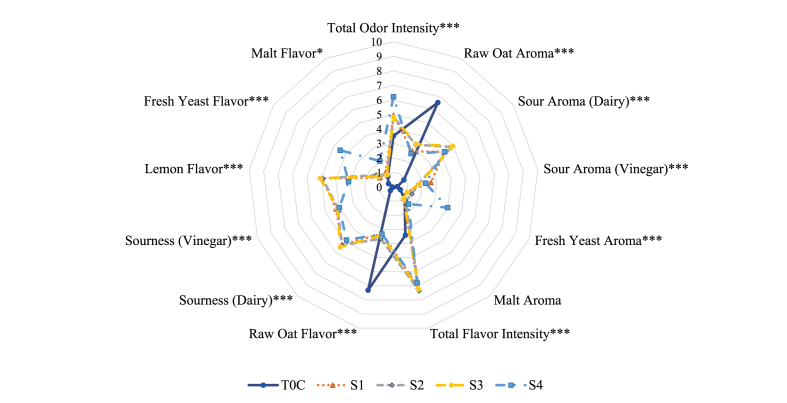
The aroma and flavor profile of T0C and sourdoughs were obtained via sensory analysis. T0C = untreated oat dough; S1 = fermented by *L. brevis* IC9; S2 = fermented by *L. plantarum* 1MR20; S3 = fermented by *L. brevis* IC9 + *L. plantarum* 1MR20, and S4 = fermented by *L. brevis* IC9 + *L. plantarum* 1MR20 + *S. cerevisiae* LNE10. Results are reported as mean scores. The number of asterisks indicate different levels of statistical significance found for ANOVA, replaced by Welch test significance in case of inhomogeneous variances. “*” = *p <* 0.05 = statistically significance; “**” = *p <* 0.01 = highly statistically significance; “***” = *p* < 0.001 = very highly statistically significance. (For interpretation of the references to color in this figure legend, the reader is referred to the Web version of this article.)

**Fig. 3:**
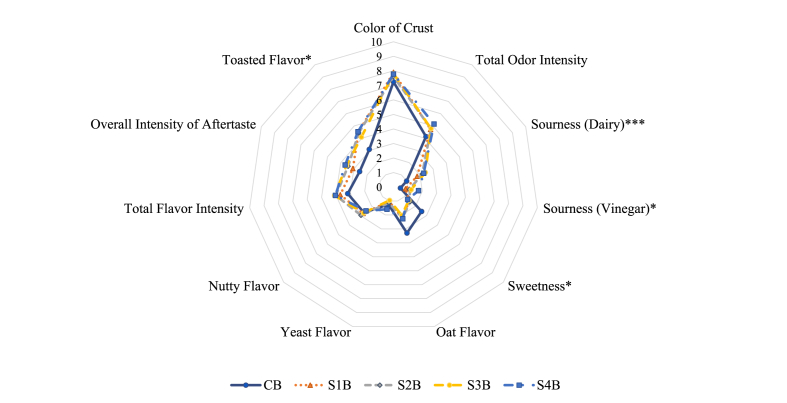
The color of crust and aroma, and flavor profile of breads obtained via sensory analysis. CB = control bread, no sourdough added; S1 = fermented by *L. brevis* IC9; S2 = fermented by *L. plantarum* 1MR20; S3 = fermented by *L. brevis* IC9 + *L. plantarum* 1MR20, and S4 = fermented by *L. brevis* IC9 + *L. plantarum* 1MR20 + *S. cerevisiae* LNE10. “B” which follows the sourdough names indicates “bread”, e.g., S1B is the bread containing S1 sourdough. The number of asterisks indicate different levels of statistical significance found for ANOVA, replaced by Welch test significance in case of inhomogeneous variances. “*” = *p* < 0.05 = statistically significance; “**” = *p* < 0.01 = highly statistically significance; “***” = *p* < 0.001 = very highly statistically significance. (For interpretation of the references to color in this figure legend, the reader is referred to the Web version of this article.)

The sensory profiles of sourdoughs and breads are displayed in Figs. 2 and 3, respectively. Mean scores, standard deviations, and ANOVA results are shown in Table A4. T0C (time 0, unfermented dough) was significantly different from other samples except for fresh yeast aroma and flavor, and malt flavor attributes. Sourdough combinations (S1, S2, S3, and S4), were discriminated from T0C, which was evaluated with higher scores of raw oat aroma and flavor, and lower scores for other attributes, such as dairy sour aroma (0.86 vs. 4.26–4.97), vinegar sour aroma (0.27 vs. 1.73–2.6), and total flavor intensity (3.41 vs. 6.79–7.36). Among the sourdoughs, when *L. brevis* IC9 and *L. plantarum* 1MR20 were used alone (S1, S2) or as a consortium (S3) they were characterized by similar aroma and flavor profiles, and no significant differences were found between these samples. However, the combination of these two LAB with the yeast (S4) conferred the sourdough distinct aroma and flavor attributes, with higher total odor intensity, fresh yeast aroma and flavor, and less lemon flavor. *L. plantarum* 1MR20 sourdough (S2) had the highest total flavor intensity (7.36). T0C had the lowest malt flavor score, while S1, S2, and S3 were similar and S4 was evaluated as the most malty sourdough (2.02).

About breads, some significant differences were found (Fig. 3; Table A4). CB was evaluated as the sweetest and with the least total odor intensity and sourness (sour flavor) as dairy and vinegar. Among the sourdough breads, the bread containing sourdough fermented by the combination of LAB and yeast (S4B) obtained the highest total odor intensity (5.15) and vinegar sourness (1.74). Moreover, S1B, S2B, and S3B were not significantly different in the evaluation of all attributes, except for dairy sourness that distinguished S1B (1.78) and all other sourdough breads (2.17–2.41), of which S3B had the highest score (2.41). Sourdough breads were significantly sourer (dairy sourness) than CB (0.99 vs. 1.78–2.41).

### 3.5 Effect of volatile compounds on sensory attributes

**Fig. 4:**
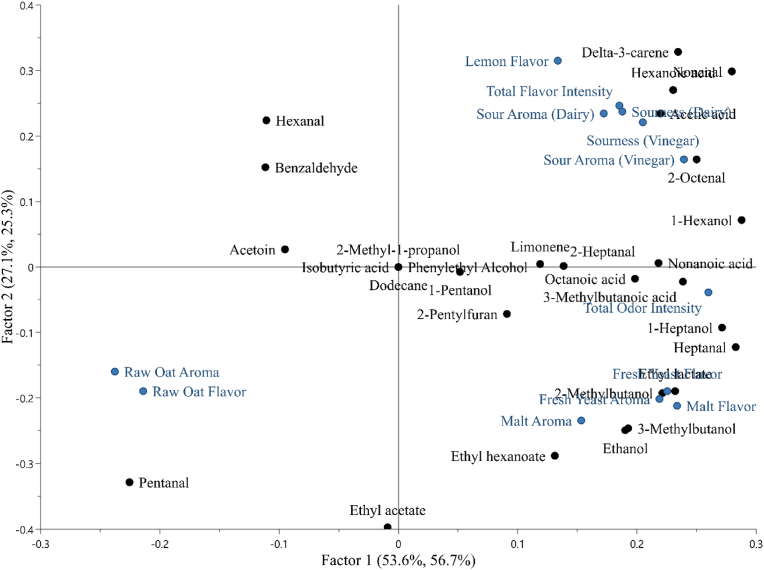
Partial least squares (PLS) regression loading plots for volatile compounds (predictors, X) and sensory attributes (responses, Y) in sourdoughs. Volatile compounds are shown as black dots and sensory attributes are represented by blue dots. Factors 1 and 2 are shown as x- and y-axes, respectively. The two percentages in parentheses next to the axis label indicate the total variance explained by the component for predictors and responses, respectively. (For interpretation of the references to color in this figure legend, the reader is referred to the Web version of this article.)

**Fig. 5:**
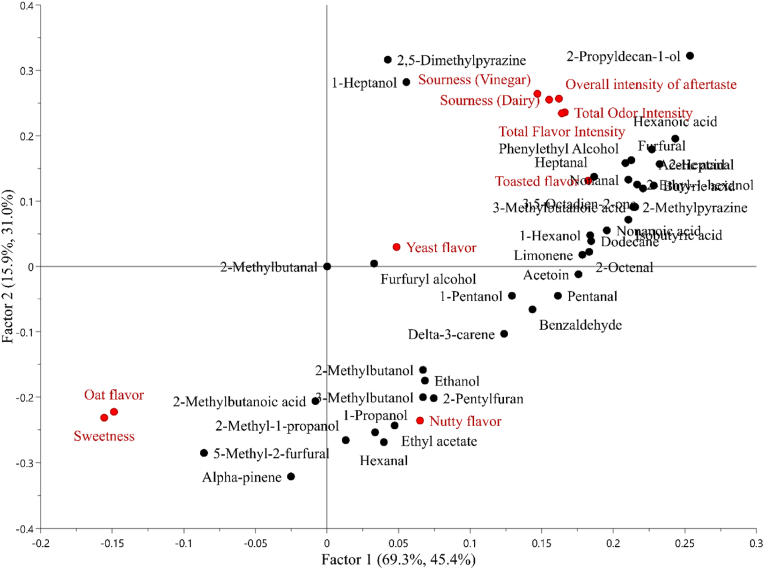
Partial least squares (PLS) regression loading plots for volatile compounds (predictors, X) and sensory attributes (responses, Y) in breads. Volatile compounds are shown as black dots and sensory attributes are represented by red dots. Factors 1 and 2 are shown as x- and y-axes, respectively. The two percentages in parentheses next to the axis label indicate the total variance explained by the component for predictors and responses, respectively. (For interpretation of the references to color in this figure legend, the reader is referred to the Web version of this article.)

Partial least squares regression (PLS) analysis was performed to investigate the effect of VOCs on sensory attributes in sourdoughs and breads (Figs. 4 and 5). In the PLS model for sourdoughs (Fig. 4), 53.6% of the variation in the VOCs explained 56.7% of the variation in the sensory analysis data for Factor 1, whereas 27.1% of the variation of VOCs explained 25.3% of the sensory data variation for Factor 2. The attributes raw oat aroma and flavor were not associated with most of the VOCs, and they were clearly distinguished from total odor aroma and flavor, and sour aroma and flavor. The model enabled to display two different and opposite patterns. The sensory attributes of sour aroma and sourness as dairy and vinegar, total flavor intensity, and lemon flavor were associated with acetic acid, hexanoic acid, nonanal, 2-octenal, delta-3-carene, 1-hexanol, and limonene. Fresh yeast and malt aroma and flavor were strongly associated with ethyl lactate, 2- and 3-methylbutanol, ethanol, and ethyl hexanoate, whereas total odor intensity with 3-methylbutanoic acid, octanoic acid, 1-heptanol, and heptanal. Other VOCs, 2-methyl-1-propanol, isobutyric acid, phenylethyl alcohol, and dodecane were located at zero value of the model because detected in only one sample no relationship was found. In the PLS model for breads (Fig. 5), 69.3% of the variation in the VOCs data explained 45.4% of the variation in the sensory data for Factor 1, whereas 15.9% of VOCs data explained 31.0% variation of sensory data for Factor 2. Oat flavor was associated with 5-methyl-2-furfural, 2-methylbutanoic acid, and alpha-pinene. Toasted flavor was strongly associated with heptanal, 2-methylpyrazine, phenylethyl alcohol, furfural, nonanoic acid, and 1-hexanol. Similar links found for toasted flavor were defined for sourness as dairy and vinegar, total flavor and odor intensity, and overall intensity of aftertaste, which were related to phenylethyl alcohol, furfural, acetic acid, however also with 2-propyldecan-1-ol, 1-heptanol, and 2,5-dimethylpyrazine. A mild relationship was found between yeast flavor and furfuryl alcohol, while nutty flavor was strongly associated with ethyl acetate, hexanal, 2-penthylfuran, 3-methylbutanol, and 1-propanol. The compound 2-methylbutanal was located at zero value of the model because detected in only one sample no relationship was found.

## 4 Discussion

In this study, different starters and their consortia used to ferment whole-oat sourdoughs were able to modify the bread flavor profile and improve bread technological quality compared to control oat bread. The growth and acidification performance of *L. brevis* IC9, *L. plantarum* 1MR20 and *S. cerevisiae* in oat sourdoughs were in accordance with previous studies on sourdough fermented by LAB and yeasts (Arora et al., 2021; Wolter et al., 2014a) reporting 8.5 and 6.5 log CFU/g cell density respectively, and acidity values of approximately 10 mL NaOH (TTA) after 24 h (Hüttner et al., 2010). However, compared to previous report, sourdoughs in this study were fermented with higher dough yield and in presence of sprouted oat grains as source of nutrients and enzymes to counteract the effect of heat treatment (Wu and Xu, 2019). Lactic acid was the main organic acid detected reaching the highest amount in sourdoughs fermented by the homofermentative *L. plantarum* 1MR20 (S2), and *L. brevis* with *L. plantarum* (S3). Acetic acid was found in appreciable amount in sourdough fermented by the heterofermentative *L. brevis* IC9 (S1) due to the added fructose (2% dw). Sourdoughs had FQ (FQ, molar ratio between lactic acid and acetic acid) ranging from 5.8 to 33.6. Sourdoughs fermented by *L. brevis* IC9 and by the association of *L. brevis* IC9 and *L. plantarum* 1MR20 with the yeast were the closest to 5 (5.8 and 9.9, respectively). The most recommended FQ for wheat sourdough should not exceed 5, although values ranging from 0.25 to 20 have also been reported (Arora et al., 2021). However, knowledge about the ideal FQ in oat sourdough is still missing. With the exception of sourdough fermented by *L. brevis* IC9 (S1), LAB and yeast in all formulations consumed almost entirely the present free sugars (glucose, sucrose, fructose, and maltose). *L. brevis* IC9 did not use maltose, whereas fructose was extensively consumed. No decrease of β-glucan content was seen in the conditions of this study, as also confirmed by a consistent viscosity of the sourdoughs, which indicates that no major modifications occurred (Mäkelä et al., 2020). Similarly, proteolysis did not occur significantly, most likely due to the inactivation of endogenous proteases during heat treatment of oat wholegrain flour. The total FAAs content decreased, suggesting an inefficient primary proteolysis and consumption by the microbial starters (Gänzle et al., 2008). Only a few FAAs were detected in similar or higher amount than in the unfermented control, such as cysteine, γ-Aminobutyric acid, glycine, ornithine, proline, and ammonia. Among sourdoughs, the association of *L. brevis* IC9 and *L. plantarum* 1MR20 with the yeast gave the highest amount of glycine, while *L. brevis* IC9 sourdough the highest value of cysteine, two amino acids commonly used as flavor enhancers in food industry (Hou et al., 2017). The association *L.* *brevis* IC9 and *L. plantarum* 1MR20 produced the highest value of ornithine, which is responsible for the roasty note in wheat bread crust (Thiele et al., 2002). Despite the high sourdough addition in bread formulation (30.3% of the dough weight) TTA values of the breads remained quite mild (<6), similar to white wheat sourdough breads (Clarke et al., 2002) and mixed oat-wheat bread (Flander et al., 2011).

Sourdough incorporation led to 11.3% higher specific volume and softer crumb (ca. 17.5%) compared to control bread. This result was in contrast with previous study, which found a lower specific volume and higher hardness in oat sourdough bread (Wolter et al., 2014a). However, compared to the previous report, several parameters and conditions used were different in our study, such as higher sourdough dough yield, however similar addition (replacing ca. 18% vs. 20% of bread flour with fermented flour in previous study). Other possible reasons are the use of different ingredients and baking conditions.

In our study, generic descriptive sensory analysis was used for both sourdoughs and sourdough breads to reveal the effects of fermentation and baking on the profile of whole grain oat breads. As expected, fermentation significantly changed the aroma and flavor of oat sourdoughs compared to the unfermented counterpart (T0C). Among sourdoughs, the presence of LAB alone (S1, S2) or in consortium (S3) did not confer detectable differences in the sensory analysis. However, sourdough fermented by *L. brevis*, *L. plantarum*, and *S. cerevisiae* in consortium (S4) resulted in a more intense total odor, fresh yeast aroma and flavor, and malt flavor. As previously reported, sourdough fermented by consortia of LAB and yeast confers a unique and more complex flavor to wheat bread (Suo et al., 2021; Y. Yang et al., 2022). This is likely due to the synergic metabolisms of *S. cerevisiae* and *L. plantarum* that can cause the release of special aroma-active compounds of bread, in the meantime the decreased amount and consumption of unpleasant indole (Zhang et al., 2023a).

The profile of sourdoughs was reflected by the profile of breads. Sourdough breads had mild aroma and taste and were ranked with scores from mild to moderately strong (0–5). The oat bread control was distinguished from sourdough breads as having less intense aroma and flavor, but higher sweetness. Overall, similarly to sourdoughs, breads containing sourdoughs fermented by LAB alone or in consortium showed a similar profile. The bread containing the sourdough fermented by LAB and yeast (S4B) had the highest total odor intensity and vinegar sourness. Main VOCs detected in oat sourdoughs and breads belonged to alcohols, aldehydes, and carboxylic acids classes, as also found in other studies on fermented oat and wheat sourdough bread (De Luca et al., 2021; He et al., 2022). In sourdoughs fermented by *L. brevis* IC9 (S1) and by the consortium of LAB and yeast (S4) ethanol and acetic acid were the most abundant. Ethanol, which gives a strong, alcoholic, ethereal, and medicinal odor, was expected due to the presence of *S. cerevisiae* (S4). Among alcohols, 2- and 3-methylbutanol were associated with S4, explained by the ability of *S. cerevisiae* LNE10, to produce these compounds from the degradation of flour amino acids, such as leucine, via the Ehrlich pathway (Birch et al., 2014; Runguphan et al., 2021). The 3-methylbutanol odor was previously defined with alcoholic, whiskey, malt, and burnt notes, whereas 2-methylbutanol with wine and onion aroma. Although with a low peak area, also ethyl lactate (fruity odor) and 1-heptanol (chemical, green), were associated with the fermentation of LAB and yeast (S4). Acetic acid, responsible for sharp, vinegar, and sour aroma, was one of the prevalent compounds associated with *L. brevis* IC9 (S1), mainly due to the highest amount. As previously found for wheat (De Luca et al., 2021), 1-hexanol was among the main alcohol compounds in all sourdoughs, especially associated with the sourdough fermented by *L. brevis* (S1). This VOC has been described with alcohol, resin, and flower aroma descriptors. Other VOCs associated with *L. brevis* fermentation (S1) were delta-3-care (lemon, resin), limonene (lemon, orange), nonanal (fat, citrus, green), and hexanoic acid (sweat). Unfermented whole grain oat (T0C) and LAB consortium fermentation (S3) were associated only with pentanal, having almond, pungent, and malt odors. Hexanal, having fatty, grass, and sweaty notes, was the main compound detected before fermentation and the most relevant aldehyde associated with *L. plantarum* 1MR20 (S2). Previously, high amount of hexanal was also found in wheat sourdoughs fermented by *L. plantarum* (Liu et al., 2020). Hexanal deriving from lipid oxidation was also detected in gluten-free doughs, with possible influence on the formation of off-flavor in bread (Pico et al., 2017).

Sourdoughs enriched the volatile profile of breads, especially when *L. plantarum* (S2B) or the consortium of LAB and yeast (S4B) were used as starters. Breads were characterized by ethanol, hexanal, minor amounts of acetic acid, and the presence of compounds derived by the Maillard reaction. Ethanol was found in all breads, however associated mainly with S2B. The high presence of ethanol in breads was expected not only because of the metabolism of yeast and heterofermentative LAB in S1 and S4, but also because fresh baker's yeast was used in bread making. Compared to sourdoughs, acetic acid diminished in all breads and was associated with S4B, most probably due to the baking process. The increased peak area of 3-methylbutanol (alcoholic, whiskey, malt, burnt) in S1B and S4B compared to CB may be due to the enrichment of the dough with sourdough. However, the high 3-methylbutanol peak area in bread containing *L. plantarum* sourdough (S2B), did not correspond to the presence of this alcohol in its respective sourdough. It is possible that fermentation of fresh baker's yeast with *L. plantarum* 1MR20 sourdough (S2) might occur more efficiently due to the presence of specific amino acids, such as arginine, which has been found to support yeast growth by protecting cells from ethanol damage (Cheng et al., 2016). The alcohol 1-hexanol (resin, flower, green) showed higher peak area in sourdough breads than CB. Aldehydes profile was similar to what previously observed for sourdoughs, with hexanal as the main compound. In a previous study, low yeast level fermentation led to higher peak area of hexanal in bread profile, as it occurred in S1B and S2B (Nor Qhairul Izzreen et al., 2016), however the lowest peak area was found in presence of consortium of LAB (S3B). Further degradation of unsaturated fatty acids via peroxidation processes, due to autoxidation or lipoxygenase activity provoked the formation of aldehydes in breads, such as hexanal (Birch et al., 2014). In this study, the exposition to oxygen during dough mixing, proofing, fermentation, and baking, and the potential enzymatic activity of lipases produced by lactic acid bacteria may have been responsible of oat lipid degradation, and subsequent moderate higher peak area of hexanal in breads (Birch et al., 2014; Silva Lopes et al., 1999). However, more investigation should be done to confirm. Flavor profile of cooked oat was previously defined as precursor-, and heat-dependent, meaning that the higher is the temperature the more oat-like, nutty, browned, or burnt flavor is formed (McGorrin, 2019). Caramelization and Maillard reaction, occurring during heating, led to the formation of furans and pyrazines, especially furfural and 2-pentylfuran. Furfural has been linked to sweet, woody, almond, bread, and rancid descriptors, while 2-pentylfuran to fruity, green, earthy, bean, and metallic (Pétel et al., 2017). Furfural was mainly detected in sourdough breads, while 2-pentylfuran in all breads, CB included. Association of LAB and yeast sourdough bread (S4B) with furfural was defined, whereas 2-pentylfuran was associated with *L. plantarum* sourdough bread (S2B). Other VOCs detected in *L. plantarum* 1MR20 bread (S2B) with the highest peak area among sourdough breads were acetoin (butter, cream), isobutyric acid (rancid, butter, cheese), alpha-pinene (fresh, camphor, sweet, green, woody, earthy, pine), and delta-3-carene (lemon, resin). The low number of connections with VOCs found for LAB consortium (S3), were confirmed in corresponding bread (S3B).

Food flavor is the result of several conditions, such as simultaneous presence of different compounds at different amounts, therefore it is necessary to consider the VOCs profile, the sensory attributes perceived, and statistical analysis conducted as a whole (PCA, PLS). Concerning the impact of VOCs on the sensory attributes evaluated for sourdoughs (Fig. 4), the presence of more intense flavor, dairy and vinegar sour aroma and flavor, and lemon flavor was mainly associated with *L. brevis* fermentation (S1), e.g., acetic acid, delta-3-carene, whereas fresh yeast and malt aroma and flavor were associated with the consortium of LAB and yeast (S4), e.g., 2- and 3-methylbutanol, ethyl lactate, ethanol. However, consortium of LAB and yeast (S4) was the only sourdough distinguished from others in the sensory evaluation, and this may be due to the differences in perception thresholds of VOCs and the accuracy of panelists. Concerning breads (Fig. 5), VOCs as ethanol and 2- and 3-methylbutanol, associated with *L. plantarum* sourdough bread (S2B), were linked to nutty flavor in the PLS model. While the total odor and flavor, sour aroma and flavor, toasted flavor, and overall intensity of aftertaste were associated with several VOCs deriving from Maillard reaction, e.g., furfural, 2-methylpyrazine, and acetic acid, alcohol, and aldehyde compounds. Most of these VOCs were associated with consortium of LAB and yeast sourdough bread (S4B). Indeed, despite *L. plantarum* S2B had the richest volatile profile, the sensory analysis did not allow to discriminate it from other sourdough breads. However, the consortium *L. brevis* IC9, *L. plantarum* 1MR20, and *S. cerevisiae* LNE10 (S4) successfully led to the differentiation of the respective bread (S4B) from other sourdough breads due to the more intense aroma and sourness.

## 5 Conclusion

This study investigated for the first time the effects of different starters on flavor development during 100% oat sourdough fermentation and baking. While oat bread properties and baking have been reported in literature, less is available on oat sourdough bread, especially concerning the effects of fermentation on flavor. Our results confirmed that sourdough addition can confer a more distinct flavor compared to common oat baking. Moreover, it was possible to modify the aroma and flavor based on the specific starter association used. Through the selection of specific consortia of lactic acid bacteria and yeasts, sourdough technology can improve the quality and sensory profile of whole-oat bread. Our research findings lay the foundation for further investigation on the liking for sourdough oat bread, via consumer study, for revealing the most appreciated sensory characteristics by consumers. This study can address consumers' preference for natural products, clean labeling, and reduced use of food additives.

## CRediT authorship contribution statement

**Silvia Cera:** Conceptualization, Data curation, Formal analysis, Investigation, Validation, Visualization, Writing – original draft, Writing – review & editing. **Fabio Tuccillo:** Conceptualization, Formal analysis, Investigation, Methodology, Validation, Visualization, Writing – review & editing. **Antti Knaapila:** Conceptualization, Methodology, Supervision, Writing – review & editing. **Finlay Sim:** Investigation. **Jessica Manngård:** Investigation. **Katariina Niklander:** Investigation. **Michela Verni:** Formal analysis, Investigation, Writing – review & editing. **Carlo Giuseppe Rizzello:** Investigation, Writing – review & editing. **Kati Katina:** Conceptualization, Supervision, Writing – review & editing. **Rossana Coda:** Conceptualization, Funding acquisition, Methodology, Project administration, Supervision, Validation, Writing – review & editing.

## Declaration of competing interest

The authors declare that they have no known competing financial interests or personal relationships that could have appeared to influence the work reported in this paper.

## Data Availability

Data will be made available on request.
