# Supplementary material for: Role of tailored sourdough fermentation in the flavor of wholegrain-oat bread
Source: Curr Res Food Sci. 2024 Feb 12;8:100697. doi: 10.1016/j.crfs.2024.100697 (PMC10937307; doi:10.1016/j.crfs.2024.100697)
Supplement: Multimedia component 1 [file mmc1.docx]

Appendix A: Supplementary material

# Sensory evaluation

- 1. **Panel performance**

A three-way ANOVA was conducted (Table A.2) to evaluate panelists' performance. The results indicated that the participants were able to distinguish among samples of sourdoughs, with statistical significance observed for 12 of 13 attributes; for the bread sensory, statistical significance was found for 6 of 11 attributes. The analysis showed statistical significance for factor Participant for almost all attributes of sourdoughs (11/13) and for all attributes of breads (11/11). For factor Sample*Participant, significance was detected for 12 of 13 attributes for sourdoughs, for 6 of 11 attributes for breads. For factor Replicate, a significant difference was detected for 5 attributes of 13 for sourdoughs and only one for breads. About Sample*Replicate interaction, one significant difference was found among 13 sourdough attributes, and no significance among 11 attributes for breads. Finally, for Replicate*Participant interaction, 11 of 13 attributes for sourdoughs, whereas 10 of 11 attributes for breads showed statistical significance.

- 1. **Distribution of data and homogeneity of variances**

Kolmogorov-Smirnova and Shapiro-Wilk tests were carried out and used to assess the deviation of data population distribution from normality (Table A.3). According to standard practices in sensory data sets, for all attributes evaluated, except for total odor intensity, data population was not normally distributed. However, skewness test showed approximate normal distribution with values ranging from -1 to 1 for all attributes, except for vinegar sour aroma, fresh yeast aroma, fresh yeast flavor, and malt flavor for sourdoughs and color of crust, vinegar sourness, sweetness, and oat and yeast flavor for breads. For sourdoughs, Kurtosis test showed values close to 3 for fresh yeast aroma, malt aroma and malt flavor, whereas in other attributes values were lower than 3. For breads, Kurtosis gave values greater than 3 for color of crust, vinegar sourness, and yeast flavor, whereas for other attributes results were lower 3. Regarding the homogeneity of variances, Levene’s test showed statistical significance for several attributes (9/11) of sourdoughs, and for two attributes of breads, sourness (vinegar) and sweetness.

# Tables

**Table 1.** Individual free amino acids (FAA) concentration in T0C and sourdoughs (n=2), reported as mg of amino acid/kg of freeze-dried sample.

|  | **T0C** | | **S1** | | **S2** | | **S3** | | **S4** | |
| --- | --- | --- | --- | --- | --- | --- | --- | --- | --- | --- |
|  | **Mean** | **Std. Dev.** | **Mean** | **Std. Dev.** | **Mean** | **Std. Dev.** | **Mean** | **Std. Dev.** | **Mean** | **Std. Dev.** |
| **Asp** | 202.91 | 3.95 | 180.22 | 14.97 | 57.32 | 6.43 | 64.28 | 0.33 | 66.02 | 13.3 |
| **Thr** | 76.58 | 0.57 | 16.78 | 0.94 | 17.75 | 1.84 | 15.63 | 2.26 | 18.48 | 2.7 |
| **Ser** | 104.29 | 0.1 | 45.41 | 1.44 | 19.42 | 2.67 | 18.48 | 1.22 | 28.81 | 5.06 |
| **Asn** | 572.75 | 25.85 | 182.67 | 16.74 | 254.45 | 20.15 | 256.22 | 1.18 | 20.33 | 4.43 |
| **Glu** | 500.45 | 10.01 | 241.46 | 25.8 | 269.32 | 25.6 | 245.44 | 5.27 | 291.41 | 39.66 |
| **Gly** | 67.99 | 1.3 | 80.48 | 5.33 | 51.94 | 4.93 | 58.68 | 0.04 | 81.98 | 10.3 |
| **Ala** | 159.6 | 1.33 | 19.45 | 0.02 | 41.4 | 4.36 | 33.01 | 0.56 | 70.8 | 12.13 |
| **Val** | 140.26 | 0.63 | 37.4 | 14.91 | 42.9 | 4.01 | 39.57 | 2.24 | 45.27 | 9.24 |
| **Cys** | 11.83 | 1.15 | 55.31 | 0.57 | 12.23 | 1.73 | 8.59 | 8.29 | 14.78 | 0.37 |
| **Met** | 30.24 | 1.15 | 9.26 | 0.07 | 4.63 | 1.01 | 26.11 | 0.76 | 30.12 | 5.53 |
| **Ile** | 68.37 | 3.3 | 14.02 | 0.64 | 15.18 | 1.56 | 17.19 | 1.61 | 20.36 | 1 |
| **Leu** | 122.25 | 3.75 | 22.47 | 1.51 | 21.37 | 3.66 | 17.38 | 3.49 | 6.82 | 0.84 |
| **Tyr** | 79.8 | 10.02 | 8.04 | 2.67 | 12.24 | 2.93 | 8.13 | 1.67 | 11.11 | 0.41 |
| **Phe** | 81.92 | 5.64 | 22.53 | 0.49 | 15.14 | 2.32 | 11.31 | 1.96 | 9.15 | 1.97 |
| **GABA** | 116.03 | 0.75 | 147.52 | 6.02 | 102.26 | 8.46 | 120.94 | 3.05 | 131.94 | 20.58 |
| **Amm** | 73.92 | 2.02 | 135.1 | 6.49 | 55.63 | 6 | 84.33 | 0.39 | 86.18 | 13.31 |
| **Orn** | 22.62 | 1.76 | 48.24 | 2.93 | 24.39 | 4.98 | 113.02 | 1.92 | 90.8 | 15.34 |
| **Lys** | 106.88 | 1.3 | 8.96 | 1.48 | 52.07 | 5.75 | 47.14 | 2.31 | 24.22 | 3.37 |
| **His** | 68.94 | 1.75 | 36.26 | 3.27 | 44.94 | 5.66 | 33.67 | 0.31 | 28.62 | 4.72 |
| **Trp** | 69.61 | 0.13 | 29.53 | 4.37 | 42.07 | 6.24 | 36.32 | 3.15 | 26.95 | 3.1 |
| **Arg** | 174.54 | 8.75 | 5.36 | 1.82 | 148.67 | 7.7 | 9.3 | 0.01 | 20.41 | 3.23 |
| **Pro** | 178.68 | 5.78 | 108.51 | 9.4 | 149.12 | 9.53 | 152.77 | 0.23 | 187.12 | 31.23 |
| **Total** | 3030.4 | 20.82 | 1454.94 | 121.87 | 1454.38 | 158.9 | 1417.43 | 25.94 | 1311.64 | 201.08 |

**Table 2.** Three-way ANOVA to evaluate panel performance in assessing the attributes intensities of oat sourdoughs and breads. *Sample* was set as fixed factor, *Replicate* and *Participant* as random factors. Values in bold indicate statistical significance (α = 0.05).

|  | **Sample** | |  | **Replicate** | |  | **Participant** | |  | **Sample*Replicate** | |  | **Sample*Participant** | |  | **Replicate*Participant** | | |
| --- | --- | --- | --- | --- | --- | --- | --- | --- | --- | --- | --- | --- | --- | --- | --- | --- | --- | --- |
|  | **F** | ***p-value*** |  | **F** | ***p-value*** |  | **F** | ***p-value*** |  | **F** | ***p-value*** |  | **F** | ***p-value*** |  | | **F** | ***p-value*** |
| **Attribute​ (Sourdoughs)** |  |  |  |  |  |  |  |  |  |  |  |  |  |  |  | |  |  |
| Total Odor Intensity​ | 7.81 | **<.001** |  | 6.343 | **0.021** |  | 4.407 | **<.001** |  | 0.566 | 0.803 |  | 2.484 | **<.001** |  | | 1.822 | **0.035** |
| Raw Oat Aroma | 26.098 | **<.001** |  | 0.383 | 0.689 |  | 2.239 | **0.042** |  | 0.392 | 0.922 |  | 2.675 | **<.001** |  | | 4.277 | **<.001** |
| Sour Aroma Dairy | 13.592 | **<.001** |  | 0.65 | 0.539 |  | 5.467 | **<.001** |  | 1.851 | 0.081 |  | 2.01 | **0.004** |  | | 2.027 | **0.016** |
| Sour Aroma Vinegar | 5.902 | **0.002** |  | 0.251 | 0.781 |  | 1.997 | 0.062 |  | 2.533 | **0.017** |  | 3.22 | **<.001** |  | | 2.399 | **0.004** |
| Fresh Yeast Aroma | 9.002 | **<.001** |  | 2.124 | 0.191 |  | 1.037 | 0.431 |  | 0.579 | 0.792 |  | 8.633 | **<.001** |  | | 1.538 | 0.097 |
| Malt Aroma | 1.204 | 0.367 |  | 2.062 | 0.164 |  | 7.951 | **<.001** |  | 1.504 | 0.17 |  | 1.374 | 0.117 |  | | 2.362 | **0.004** |
| Total Flavor Intensity | 20.385 | **<.001** |  | 3.7 | **0.048** |  | 3.95 | **<.001** |  | 1.077 | 0.388 |  | 5.597 | **<.001** |  | | 3.59 | **<.001** |
| Raw Oat Flavor | 44.172 | **<.001** |  | 6.826 | **0.017** |  | 7.972 | **<.001** |  | 0.315 | 0.958 |  | 2.24 | **0.001** |  | | 2.265 | **0.006** |
| Sourness (Dairy) | 37.722 | **<.001** |  | 4.756 | **0.037** |  | 8.456 | **<.001** |  | 0.845 | 0.567 |  | 5.03 | **<.001** |  | | 1.731 | **0.049** |
| Sourness (Vinegar) | 35.359 | **<.001** |  | 1.203 | 0.344 |  | 7.028 | **<.001** |  | 0.628 | 0.751 |  | 2.685 | **<.001** |  | | 1.864 | **0.03** |
| Lemon Flavor | 42.275 | **<.001** |  | 6.23 | **0.018** |  | 3.595 | **0.003** |  | 1.347 | 0.234 |  | 2.586 | **<.001** |  | | 1.514 | 0.105 |
| Fresh Yeast Flavor | 10.378 | **<.001** |  | 1.12 | 0.351 |  | 2.164 | **0.038** |  | 1.676 | 0.118 |  | 6.33 | **<.001** |  | | 2.93 | **<.001** |
| Malt Flavor | 5.047 | **0.003** |  | 3.282 | 0.064 |  | 11.267 | **<.001** |  | 1.215 | 0.302 |  | 3.617 | **<.001** |  | | 3.237 | **<.001** |
|  |  |  |  |  |  |  |  |  |  |  |  |  |  |  |  | |  |  |
| **Attribute​ (Breads)** |  |  |  |  |  |  |  |  |  |  |  |  |  |  |  | |  |  |
| Color of Crust | 7.307 | **0.01** |  | 5.292 | **0.015** |  | 2.676 | **0.036** |  | 1.348 | 0.234 |  | 1.095 | 0.365 |  | | 10.514 | **<.001** |
| Total Odor Intensity​ | 20.703 | 0.067 |  | 0.306 | 0.74 |  | 2.832 | **0.031** |  | 0.549 | 0.816 |  | 0.792 | 0.776 |  | | 9.297 | **<.001** |
| Sourness (Dairy) | 10.188 | **<.001** |  | 3.562 | 0.085 |  | 8.683 | **<.001** |  | 0.559 | 0.808 |  | 1.705 | **0.028** |  | | 1.63 | 0.075 |
| Sourness (Vinegar) | 3.634 | **0.03** |  | 1.076 | 0.375 |  | 4.476 | **0.002** |  | 1.064 | 0.398 |  | 1.829 | **0.015** |  | | 1.855 | **0.034** |
| Sweetness | 8.241 | **<.001** |  | 2.877 | 0.085 |  | 5.704 | **<.001** |  | 1.188 | 0.318 |  | 2.353 | **0.001** |  | | 4.009 | **<.001** |
| Oat Flavor | 13.124 | **0.028** |  | 0.419 | 0.665 |  | 14.569 | **<.001** |  | 0.925 | 0.501 |  | 0.723 | 0.856 |  | | 4.257 | **<.001** |
| Yeast Flavor | 1.896 | 0.174 |  | 0.425 | 0.66 |  | 12.787 | **<.001** |  | 1.217 | 0.302 |  | 1.62 | **0.042** |  | | 4.932 | **<.001** |
| Nutty Flavor | 1.483 | 0.4 |  | 1.64 | 0.233 |  | 9.896 | **<.001** |  | 0.653 | 0.731 |  | 0.832 | 0.724 |  | | 2.972 | **<.001** |
| Total Flavor Intensity | 3.001 | 0.052 |  | 0.713 | 0.505 |  | 5.702 | **<.001** |  | 1.035 | 0.418 |  | 1.926 | **0.009** |  | | 4.877 | **<.001** |
| Overall Intensity of Aftertaste | 2.398 | 0.087 |  | 0.358 | 0.705 |  | 4.953 | **<.001** |  | 0.826 | 0.582 |  | 2.352 | **0.001** |  | | 3.266 | **<.001** |
| Toasted Flavor | 12.356 | **0.012** |  | 0.204 | 0.818 |  | 6.961 | **<.001** |  | 0.9 | 0.521 |  | 0.881 | 0.656 |  | | 5.596 | **<.001** |

**Table 3.** Skewness, Kurtosis, Kolmogorov-Smirnova (with Lilliefors significance correction), and Shapiro-Wilk statistics to assess normality of distribution and Levene statistic to assess homogeneity for each sensory attribute. Values in bold indicate statistical significance (α = 0.05).

|  | **Skewness** | **Kurtosis** | **Kolmogorov-Smirnova** | | **Shapiro-Wilk** | | **Levene** | |
| --- | --- | --- | --- | --- | --- | --- | --- | --- |
| **Attribute (Sourdoughs)** |  |  | Statistic | *p-value* | Statistic | *p-value* | Statistic | *p-value* |
| Total Odor Intensity | -0.128 | -0.865 | 0.056 | .200* | 0.976 | **0.007** | 0.374 | 0.827 |
| Raw Oat Aroma | 0.392 | -1.089 | 0.114 | **<.001** | 0.933 | **<.001** | 1.867 | 0.119 |
| Sour Aroma (Dairy) | 0.216 | -1.229 | 0.122 | **<.001** | 0.935 | **<.001** | 7.656 | **<.001** |
| Sour Aroma (Vinegar) | 1.068 | 0.674 | 0.147 | **<.001** | 0.89 | **<.001** | 8.108 | **<.001** |
| Fresh Yeast Aroma | 1.753 | 2.921 | 0.227 | **<.001** | 0.76 | **<.001** | 16.342 | **<.001** |
| Malt Aroma | 1.603 | 2.632 | 0.169 | **<.001** | 0.834 | **<.001** | 1.598 | 0.177 |
| Total Flavor Intensity | -0.973 | 0.079 | 0.158 | **<.001** | 0.895 | **<.001** | 5.243 | **<.001** |
| Raw Oat Flavor | 0.33 | -1.139 | 0.106 | **<.001** | 0.938 | **<.001** | 0.435 | 0.783 |
| Sourness (Dairy) | -0.079 | -1.163 | 0.1 | **<.001** | 0.938 | **<.001** | 9.287 | **<.001** |
| Sourness (Vinegar) | 0.035 | -1.449 | 0.137 | **<.001** | 0.9 | **<.001** | 22.292 | **<.001** |
| Lemon Flavor | -0.034 | -1.185 | 0.1 | **<.001** | 0.937 | **<.001** | 18.041 | **<.001** |
| Fresh Yeast Flavor | 1.5 | 1.238 | 0.233 | **<.001** | 0.75 | **<.001** | 14.225 | **<.001** |
| Malt Flavor | 1.86 | 3.288 | 0.228 | **<.001** | 0.75 | **<.001** | 2.496 | **0.045** |
| **Attribute (Breads)** |  |  |  |  |  |  |  |  |
| Color of Crust | -1.693 | 4.654 | 0.126 | **<.001** | 0.869 | **<.001** | 0.213 | 0.931 |
| Total Odor Intensity | -0.159 | -0.054 | 0.066 | .200* | 0.991 | 0.433 | 0.236 | 0.918 |
| Sourness (Dairy) | 0.767 | -0.042 | 0.116 | **<.001** | 0.938 | **<.001** | 1.179 | 0.323 |
| Sourness (Vinegar) | 2.613 | 8.367 | 0.225 | **<.001** | 0.711 | **<.001** | 2.936 | **0.023** |
| Sweetness | 1.313 | 2.3 | 0.112 | **<.001** | 0.899 | **<.001** | 4.346 | **0.002** |
| Oat Flavor | 1.011 | 0.379 | 0.127 | **<.001** | 0.898 | **<.001** | 0.307 | 0.873 |
| Yeast Flavor | 2.27 | 4.849 | 0.267 | **<.001** | 0.674 | **<.001** | 0.616 | 0.652 |
| Nutty Flavor | 0.646 | -0.349 | 0.126 | **<.001** | 0.947 | **<.001** | 0.143 | 0.966 |
| Total Flavor Intensity | 0.402 | -0.568 | 0.084 | **0.011** | 0.97 | **0.002** | 0.86 | 0.489 |
| Overall Intensity of Aftertaste | 0.951 | 0.555 | 0.092 | **0.003** | 0.928 | **<.001** | 1.295 | 0.275 |
| Toasted Flavor | 0.34 | -1.084 | 0.099 | **0.001** | 0.944 | **<.001** | 0.595 | 0.667 |

* This is a lower bound of the true significance.

**Table 4.** Sensory analyses data from sourdoughs (n=32) and breads (n=30). F values (ANOVA), significance (ANOVA), averages and standard deviations of samples evaluated. Different superscript lowercase letters (a-c) in the same row indicate statistically significant (Tukey’s, *p* < 0.05) differences.

| **Attribute (Sourdoughs)** | **F** | **Sig** | **T0C** | **S1** | **S2** | **S3** | **S4** |
| --- | --- | --- | --- | --- | --- | --- | --- |
| Total Odor Intensity | 6.81 | **<0.001** | 3.52 ± 1.83^a^ | 4.94±2.06^ab^ | 4.83±2.28^ab^ | 4.80±2.22^ab^ | 6.21±1.93^b^ |
| Raw Oat Aroma | 20.54 | **<0.001** | 6.57 ±1.74^b^ | 2.78±1.97^a^ | 3.28±2.04^a^ | 3.33±2.39^a^ | 2.60±1.96^a^ |
| Sour Aroma (Dairy)^*^ | 34.1 | **<0.001** | 0.86±1.22^a^ | 4.26±2.49^b^ | 4.87±2.81^b^ | 4.97±2.65^b^ | 4.28±2.53^b^ |
| Sour Aroma (Vinegar)^*^ | 28.21 | **<0.001** | 0.27±0.52^a^ | 2.60±1.88^b^ | 1.73±1.39^b^ | 1.79±1.34^b^ | 2.23±1.75^b^ |
| Fresh Yeast Aroma^*^ | 11.25 | **<0.001** | 0.51±0.96^a^ | 0.95±1.36^a^ | 1.35±1.32^a^ | 1.01±1.29^a^ | 3.98±2.84^b^ |
| Malt Aroma | 0.95 | 0.44 | 1.16±1.58^a^ | 1.03±1.08^a^ | 1.25±1.12^a^ | 1.05±1.07^a^ | 1.56±1.37^a^ |
| Total Flavor Intensity^*^ | 16.18 | **<0.001** | 3.41±2.51^a^ | 7.05±1.68^b^ | 7.36±1.39^b^ | 7.23±1.59^b^ | 6.79±2.10^b^ |
| Raw Oat Flavor | 20.99 | **<0.001** | 7.31±2.00^b^ | 3.31±2.26^a^ | 3.66±2.28^a^ | 3.40±2.07^a^ | 3.33±2.16^a^ |
| Sourness (Dairy)^*^ | 87.96 | **<0.001** | 0.33±0.90^a^ | 5.30±2.23^b^ | 5.36±2.34^b^ | 5.54±2.38^b^ | 4.87±2.43^b^ |
| Sourness (Vinegar)^*^ | 103.09 | **<0.001** | 0.04±0.12^a^ | 4.23±2.22^b^ | 4.09±2.44^b^ | 4.11±2.28^b^ | 3.99±1.98^b^ |
| Lemon Flavor^*^ | 188.84 | **<0.001** | 0.08±0.19^a^ | 4.93±1.87^c^ | 4.93±1.70^c^ | 5.09±1.73^c^ | 3.12±2.05^b^ |
| Fresh Yeast Flavor^*^ | 17 | **<0.001** | 0.42±0.60^a^ | 1.14±1.77^a^ | 1.45±1.98^a^ | 1.27±1.85^a^ | 4.46±2.91^b^ |
| Malt Flavor^*^ | 2.6 | **0.043** | 0.83±0.96^a^ | 1.15±1.85^ab^ | 0.97±1.49^ab^ | 0.96±1.36^ab^ | 2.02±1.87^b^ |
|  |  |  |  |  |  |  |  |
| **Attribute (Breads)** | **F** | **Sig** | **CB** | **S1B** | **S2B** | **S3B** | **S4B** |
| Color of Crust | 2.24 | 0.07 | 7.24±1.12^a^ | 7.90±0.71^a^ | 7.61±1.04^a^ | 7.81±0.87^a^ | 7.77±0.97^a^ |
| Total Odor Intensity | 2.01 | 0.1 | 4.13±1.56^a^ | 4.72±1.42^ab^ | 4.77±1.32^ab^ | 4.78±1.40^ab^ | 5.15±1.37^b^ |
| Sourness (Dairy) | 5.21 | **0.001** | 0.99±1.38^a^ | 1.78±1.10^ab^ | 2.17±1.45^b^ | 2.41±1.42^b^ | 2.28±1.49^b^ |
| Sourness (Vinegar)^*^ | 3.14 | **0.02** | 0.48±1.03^a^ | 0.84±0.76^ab^ | 1.12±1.45^ab^ | 1.23±1.42^ab^ | 1.74±1.97^b^ |
| Sweetness^*^ | 3.23 | **0.02** | 2.56±1.74^b^ | 1.42±1.02^a^ | 1.52±1.17^a^ | 1.39±1.01^a^ | 1.29±0.90^a^ |
| Oat Flavor | 1.74 | 0.14 | 3.30±2.24^a^ | 2.27±2.07^a^ | 2.21±1.99^a^ | 2.02±1.95^a^ | 2.27±2.20^a^ |
| Yeast Flavor | 0.58 | 0.68 | 1.24±1.81^a^ | 1.04±1.51^a^ | 1.13±1.88^a^ | 0.95±1.61^a^ | 1.59±2.02^a^ |
| Nutty Flavor | 0.25 | 0.91 | 2.69±1.92^a^ | 2.77±1.86^a^ | 2.96±1.84^a^ | 2.73±1.74^a^ | 2.50±1.72^a^ |
| Total Flavor Intensity | 1.57 | 0.18 | 3.18±1.55^a^ | 3.70±1.57^a^ | 4.01±1.78^a^ | 4.10±1.49^a^ | 4.04±1.90^a^ |
| Overall Intensity of Aftertaste | 1.76 | 0.14 | 2.56±1.51^a^ | 3.07±1.77^a^ | 3.44±1.87^a^ | 3.56±1.75^a^ | 3.66±2.24^a^ |
| Toasted Flavor | 2.51 | **0.04** | 3.08±1.85^a^ | 4.46±2.16^a^ | 4.43±2.22^a^ | 4.09±2.13^a^ | 4.53±2.03^a^ |

* Due to inhomogeneous variances, ANOVA’s F statistic values and significance were replaced with Welch test’s statistics.
